# Higher-order thalamocortical circuits are specified by embryonic cortical progenitor types in the mouse brain

**DOI:** 10.1016/j.celrep.2024.114157

**Published:** 2024-04-26

**Authors:** Matthew J. Buchan, Gemma Gothard, Kashif Mahfooz, Joram J. van Rheede, Sophie V. Avery, Alexios Vourvoukelis, Alexander Demby, Tommas J. Ellender, Sarah E. Newey, Colin J. Akerman

**Affiliations:** 1 Department of Pharmacology, Mansfield Road, OX1 3QT Oxford, UK; 2 Experimental Neurobiology Unit, Universiteitsplein, 2610 Antwerp, Belgium

## Abstract

The sensory cortex receives synaptic inputs from both first-order and higher-order thalamic nuclei. First-order inputs relay simple stimulus properties from the periphery, whereas higher-order inputs relay more complex response properties, provide contextual feedback, and modulate plasticity. Here, we reveal that a cortical neuron’s higher-order input is determined by the type of progenitor from which it is derived during embryonic development. Within layer 4 (L4) of the mouse primary somatosensory cortex, neurons derived from intermediate progenitors receive stronger higher-order thalamic input and exhibit greater higher-order sensory responses. These effects result from differences in dendritic morphology and levels of the transcription factor Lhx2, which are specified by the L4 neuron’s progenitor type. When this mechanism is disrupted, cortical circuits exhibit altered higher-order responses and sensory-evoked plasticity. Therefore, by following distinct trajectories, progenitor types generate diversity in thalamocortical circuitry and may provide a general mechanism for differentially routing information through the cortex.

## Graphical abstract

**Graphical abstract:**
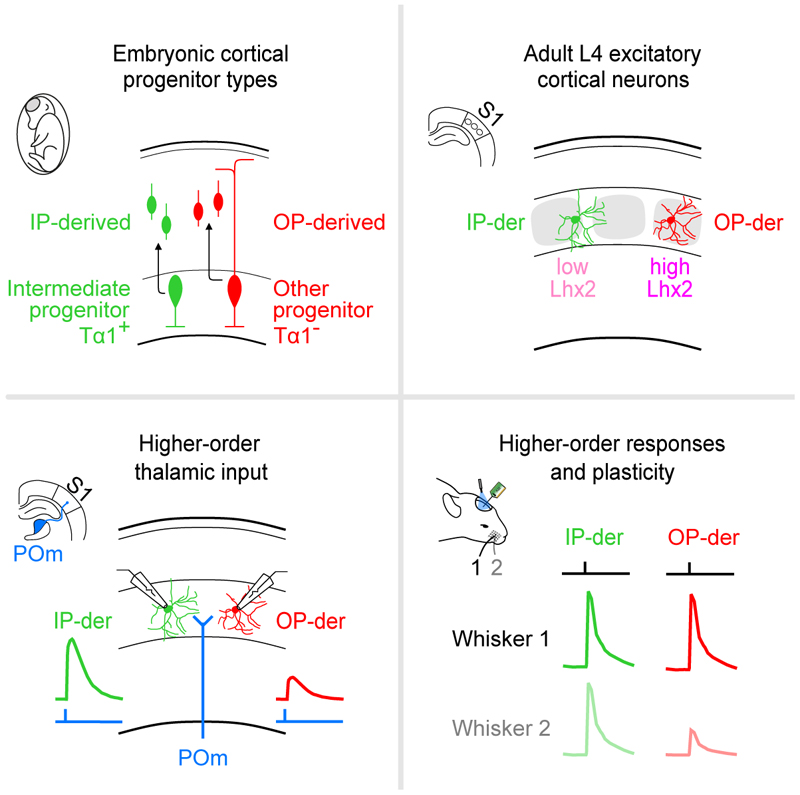

## Introduction

Cortical neurons receive and integrate synaptic inputs from different thalamic nuclei, which relay distinct types of information.^1–4^ Inputs from so-called first-order thalamic nuclei are considered the primary relay from the sensory periphery to the cortex and transfer sensory information via neurons that exhibit simple response properties.^5^ In contrast, inputs from higher-order thalamic nuclei relay information from multiple sources that can be of a cortical or subcortical origin.^6–9^ Consequently, higher-order inputs to the cortex are associated with more complex response properties, reflecting the encoding of both sensory and contextual information.^6,10^ In addition to facilitating the transfer of sensory information through an ascending hierarchy of transthalamic pathways,^11,12^ higher-order thalamic nuclei also send feedback projections to lower cortical areas, where they are thought to influence the processing and plasticity of first-order inputs.^13–16^

Even within layer 4 (L4), one of the earliest stages of cortical processing, neurons can receive both first-order and higher-order thalamic inputs. In the rodent primary somatosensory cortex (S1), tactile information from single whiskers is conveyed via neurons in the first-order, ventral posterior medial nucleus (VPM) of the thalamus. VPM projections target L4 within S1, forming anatomical structures called barrels and creating a somatotopic map of the mystacial pad.^17,18^ Meanwhile, information from multiple whiskers is conveyed via neurons within the higher-order, posterior medial nucleus (POm).^6,8^ POm projections to S1 target the inter-barrel regions within L4, called septa, as well as L1 and L5a.^19–23^ Previous work, using both electrical and optical stimulation methods, has shown that, while VPM represents the major input to L4, individual neurons differ in the degree to which they receive higher-order input from POm.^24–26^ However, how cortical neurons are specified to receive their higher-order thalamocortical input is unknown.

One potential explanation is that thalamic inputs reflect the cortical neuron’s developmental history. In the mouse, for example, all excitatory cortical neurons are born from a pool of progenitor cells that reside in the ventricular proliferative zones of the embryonic cortex.^27^ During early stages of corticogenesis, the ventricular zone is mainly populated by radial glial cells that initially undergo symmetric, proliferative divisions to amplify the radial glial cell pool.^28–30^ As neurogenesis proceeds, radial glial cells switch to undergo mainly asymmetric divisions, resulting in self-renewal and the production of either a neuron or an intermediate progenitor (IP), which can then go on to produce neurons.^29–37^ Unlike radial glial cells, neurogenic IPs exhibit a single symmetric division that produces two daughter neurons.^32,35,38,39^ IPs can be distinguished from radial glial cells and further divided into subpopulations based on their gene expression profile, morphology, and location within the germinal zone.^38–46^ These different progenitors create lineage heterogeneity so that neurons destined for the same cortical layer can be derived via different developmental trajectories.^47,48^

Here we examine whether the thalamocortical input received by an L4 neuron is related to the progenitor type from which the neuron was derived during embryonic development. By combining *in utero* labeling techniques with *in vivo* electrophysiology and optical circuit mapping, we characterize L4 neurons derived from a population of IPs (IP-derived) and compare these to L4 neurons derived from other progenitors (OP-derived). We find that the IP-derived neurons exhibit pronounced multi-whisker response properties and receive stronger input from higher-order thalamus compared to neighboring OP-derived neurons. These properties are shown to result from progenitor type-dependent differences in the molecular and morphological development of the cortical neuron, which, when altered, causes IP-derived neurons to exhibit single-whisker responses, receive weaker higher-order input, and disrupt sensory-evoked cortical plasticity. Thus, we show that thalamocortical circuits for the processing of higher-order inputs are specified by a cortical progenitor type-dependent mechanism.

## Results

### S1 L4 neurons differ in their multi-whisker response properties

**Figure 1:**
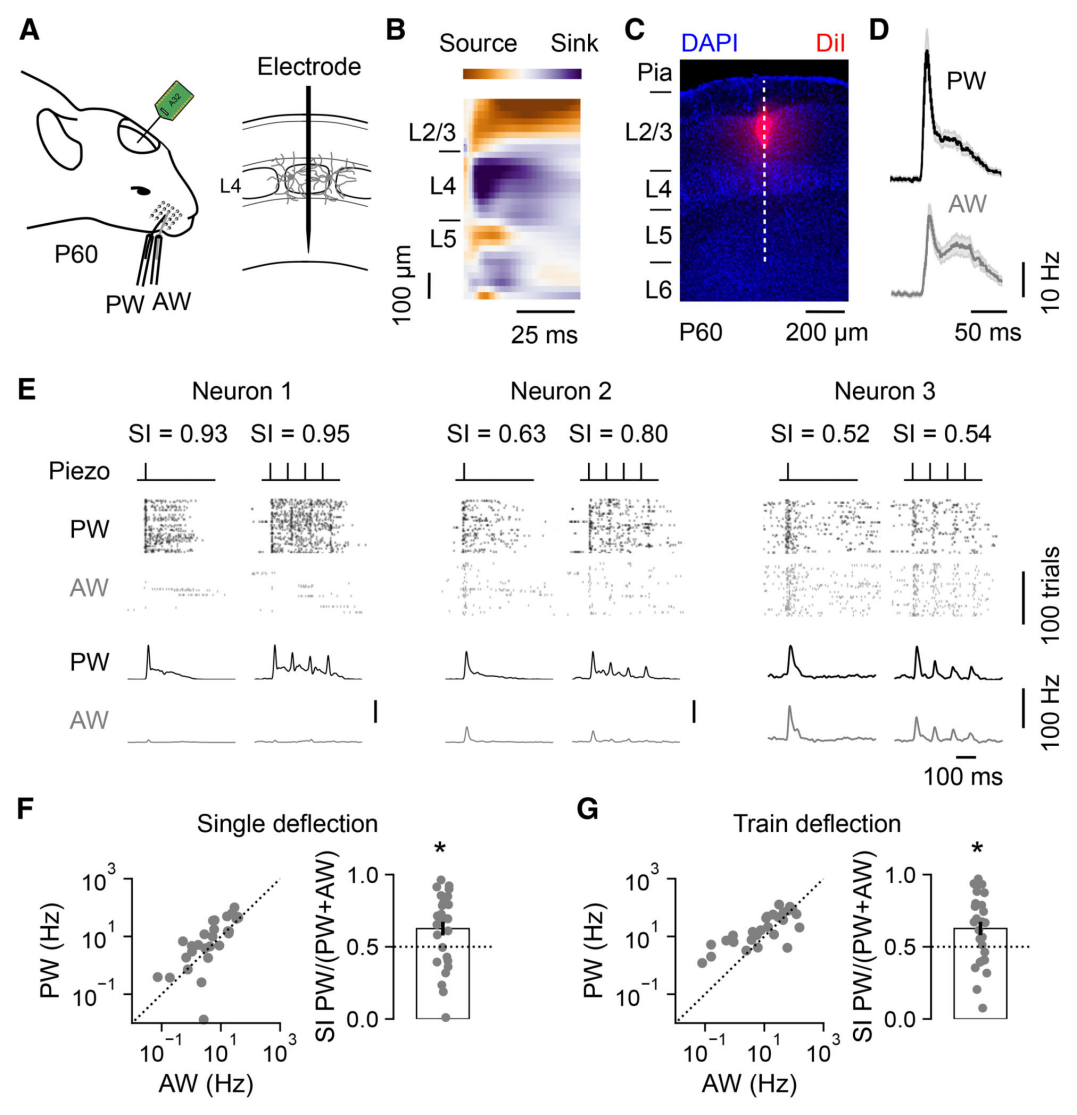
S1 L4 neurons differ in their multi-whisker response properties (A)The activity of individual regular-spiking L4 neurons in S1 was recorded in response to deflection of the PW or AW at P60. (B) L4 was identified using the current source density profile following PW deflection. (C) Histology confirmed the recording location in S1. The dashed line indicates electrode penetration. (D) Mean L4 neuronal responses from an individual animal following a single deflection of the PW (top) or AW (bottom). Shading indicates SEM around the mean. (E) Three L4 neurons with different SI, as defined by relative response to the PW and AW. Raster plots and corresponding peri-stimulus time histograms (PSTHs) show spiking activity over 100 trials of either a single deflection (inner left) or train deflection (inner right) of the PW (black) or AW (gray). (F) Responses of individual neurons to single deflection of the PW and AW (left) and the distribution of corresponding SI values (right). L4 neurons were selective for the PW (*n* = 28 neurons, *p* = 0.006, one-sample t test). (G) Responses of individual neurons to train deflection of the PW and AW (left) and the corresponding SI values (right). L4 neurons were selective for the PW (*n* = 28 neurons, *p* = 0.006, one-sample t test). Data are represented as mean ± SEM. Scale bars are indicated.

To investigate the response properties of L4 excitatory neurons in S1, we performed extracellular recordings in anesthetized mice at post-natal day 60 (P60). Responses were measured to deflections of the principal whisker (PW) and an adjacent whisker (AW; Figure 1A; STAR Methods), while regular spiking (primarily excitatory) neurons in S1 were identified based on their wave-form properties (Figure S1). L4 was identified using the current-source density profile following PW deflection (Figure 1B), and the electrode location in S1 was confirmed by post hoc histological analysis (Figure 1C). Correct identification of the PW and AW was confirmed offline from the response properties (Figure 1D; STAR Methods). Consistent with previous work, individual L4 excitatory neurons were responsive to PW and AW deflection.^49,50^

To quantify the response properties of L4 neurons, we defined a selectivity index (SI) in which larger values indicated neurons that responded mainly to the PW. SI values closer to 0.5 indicated similar levels of response to the PW and AW, consistent with higher-order response properties.^51^ We assessed the SI following either single whisker deflections or trains of whisker deflections (four deflections at 10 Hz). Some L4 excitatory neurons displayed very high SI values, whereas others displayed SI values close to 0.5, indicative of higher-order responses (Figure 1E). Consequently, while L4 excitatory neurons were more responsive to deflections of the PW (Figures 1F and 1G; single deflection mean SI was 0.61 ± 0.04; train deflection mean SI was 0.64 ± 0.04), the range of SI values revealed that individual L4 excitatory neurons exhibit heterogeneity in their response properties (single deflection interquartile range [IQR] was 0.48–0.76; train deflection IQR was 0.53–0.79). Given that thalamo-cortical information relating to multiple whiskers is conveyed via POm,^6,8^ these data are consistent with the observation that individual L4 neurons differ in the degree to which they receive higher-order thalamic input.^24–26^

### IP-derived L4 neurons exhibit multi-whisker responses

**Figure 2:**
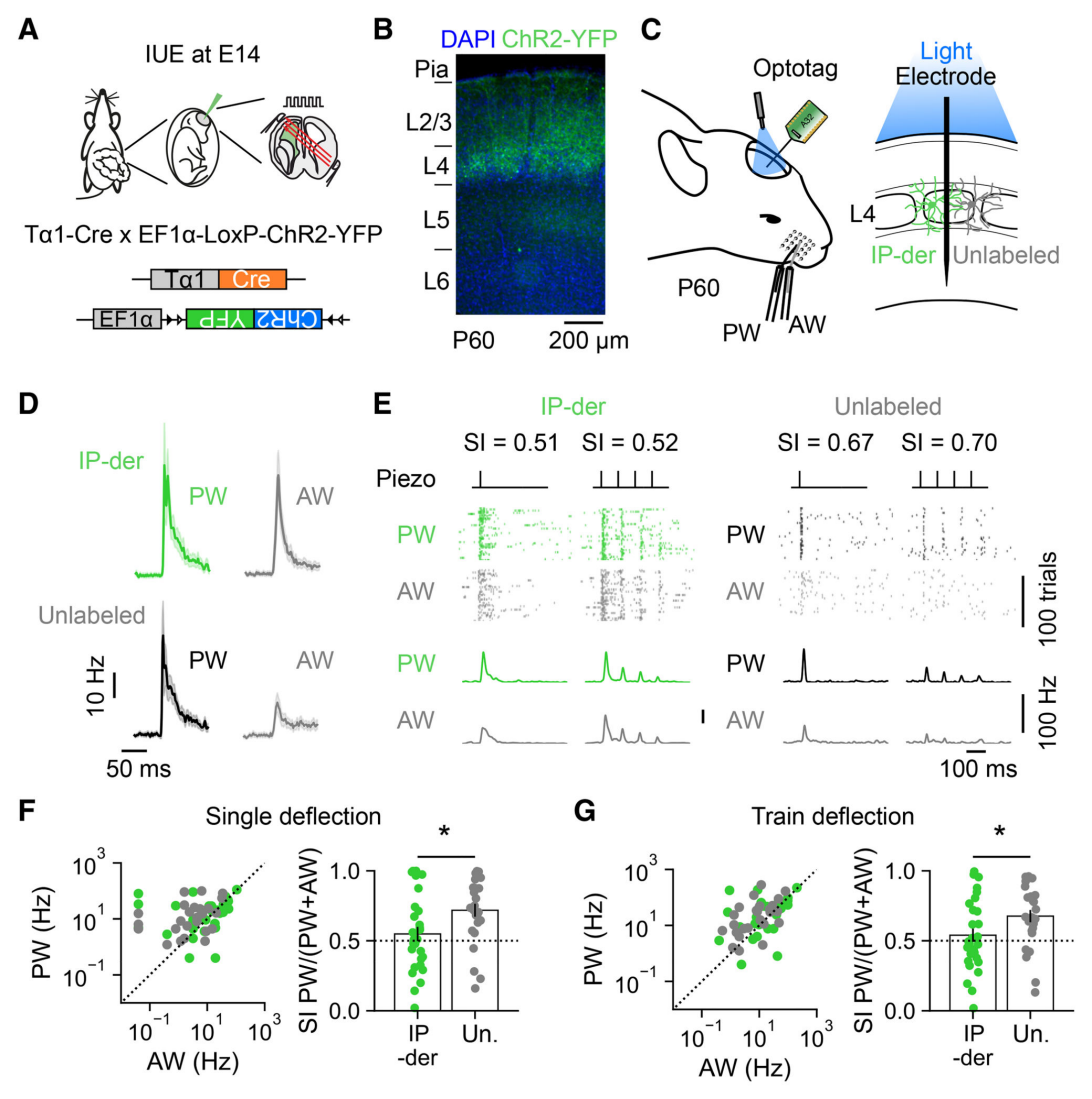
IP-derived L4 neurons exhibit greater multi-whisker responses (A) IUE of a Tα1-Cre and floxed ChR2-YFP plasmid was used to optotag IP-derived L4 neurons in S1. (B) IP-derived S1 L4 neurons expressing ChR2-YFP at P60. (C) The spiking activity of optotagged IP-derived L4 neurons and neighboring (non-optotagged) unla-beled L4 neurons was recorded in response to the deflection of the PW or AW. (D) Mean responses from an individual animal following a single deflection of the PW (left) or AW (right). (E) Spiking of an individual IP-derived (left, green) and an unlabeled L4 neuron (right, black) over 100 trials of either single deflection (inner left) or train deflection (inner right) of the PW or AW. (F) Responses of individual IP-derived and unlabeled L4 neurons to single deflection of the PW and AW (left) and the distribution of corresponding SI values (right). IP-derived L4 neurons were less selective to the PW, and therefore relatively more responsive to the AW, when compared to unlabeled neurons (*n* = 29 and 26, *p* = 0.017, t test). (G) Responses to train deflection of the PW and AW (left) and corresponding SI values (right). IP-derived L4 neurons were less selective to the PW and therefore relatively more responsive to the AW (*n* = 29 and 26, *p* = 0.040, t test). Data are represented as mean ± SEM; *n* = neurons; conventions as in Figure 1. Scale bars are indicated.

To investigate whether heterogeneity in the response properties of L4 excitatory neurons is associated with the neuron’s progenitor type, we used *in utero* electroporation (IUE; STAR Methods) to pulse label a population of dividing excitatory progenitor cells at embryonic day 14 (E14), when S1 L4 excitatory neurons are being born.^52,53^ In line with previous work, we used the tubulin alpha1 (Tα1) promoter to label IPs within the germinal zone of the embryonic cortex that gives rise to S1.^36–38^ Further characterization of this labeling strategy (Figure S2) supported the conclusion that the Tα1 promoter labels a population of IPs that includes apical IPs^38–40,46^ and basal IPs.^54^ We here refer to the neurons derived from this population of progenitors as “IP-derived.” To establish the response properties of the mature IP-derived L4 neurons in the postnatal cortex, we used optotagging as an established method for identifying cells *in vivo*^55^ (Figures 2A–2C). This strategy required us to electroporate two DNA constructs at E14: a Tα1-Cre construct in which Cre recombinase is under the control of a portion of the Tα1 promoter^39^ and a floxed ChR2-YFP reporter construct that expresses Channelr-hodopsin-2 (ChR2) fused to enhanced yellow fluorescent protein (YFP) following Cre recombination (Figure 2A). This resulted in the expression of ChR2-YFP in IP-derived L4 neurons, and the IUE was targeted so that, when the animals matured and reached P60, labeling of IP-derived ChR2-YFP-expressing neurons could be observed in L4 (Figure 2B). Having validated the optotagging method (Figure S3), neurons that exhibited reliable, short-latency (<5 ms), light-evoked responses were considered to be directly activated by ChR2 and therefore classified as “IP-derived.” Those neurons that exhibited a longer latency or no light-evoked responses were defined as “unlabeled.” As above, deflections of the PW and AW evoked responses in both the IP-derived and unlabeled L4 neurons (Figure 2D). However, compared to unlabeled neurons, IP-derived neurons showed stronger relative responses to the AW. This was captured by lower SI values for the IP-derived neurons to both single and train deflections of the whiskers (Figures 2E–2G; single deflection mean SI was 0.55 ± 0.05 for IP-derived and 0.71 ± 0.05 for unlabeled; train deflection mean SI was 0.54 ± 0.05 for IP-derived and 0.67 ± 0.04 for unlabeled). The differences in SI emerged over a timescale following whisker deflection that was consistent with previous work and the conclusion that IP-derived L4 neurons receive stronger recurrent activity via the thalamus^6,56,57^ (Figure S4). These data reveal that the IP-derived neurons tend to exhibit multi-whisker responsivity, establishing that the response properties of L4 excitatory neurons are related to the progenitor type from which the neuron is derived.

### IP-derived L4 neurons receive greater input from higher-order thalamus

**Figure 3:**
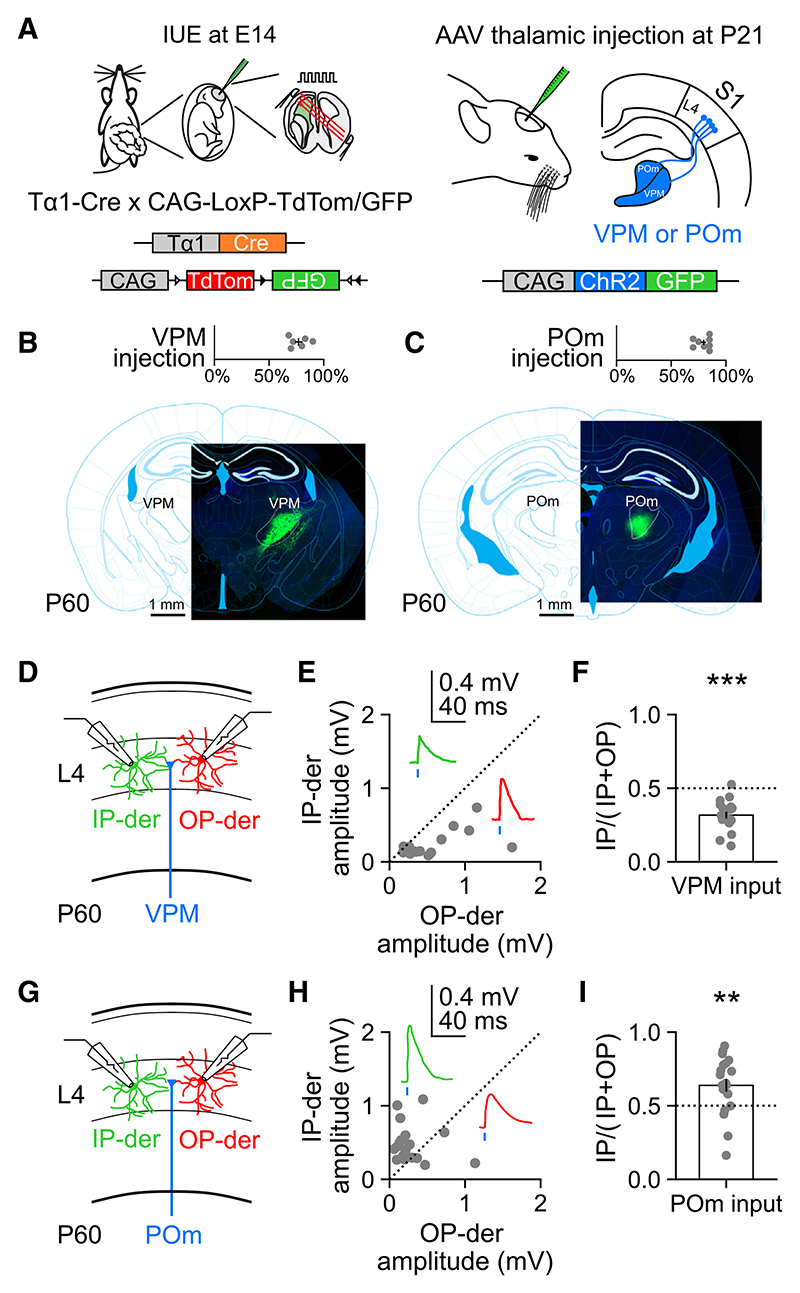
IP-derived L4 neurons receive greater input from higher-order thalamus (A) Experimental design for studying thalamic input to progenitor type-defined L4 neurons. IUE of a Tα1-Cre and two-color Cre-dependent reporter plasmid was used to label IP-derived (GFP expressing, green) and OP-derived (tdTo-mato expressing, red) L4 neurons in S1 (left). At P21, the mice received a thalamic injection of an AAV encoding CAG-ChR2-GFP into either VPM or POm (right). (B) Coronal brain slice through the thalamus at P60 in a VPM-injected animal, with a corresponding section from a brain atlas overlaid (bottom). Histological analysis revealed that 77.2% ± 3.5% (*n* = 6) of ChR2-GFP expression was restricted to VPM (top). (C) Coronal brain slice through the thalamus in a POm-injected animal, with a corresponding section from brain atlas overlaid (bottom). Histological analysis revealed that 80.4% ± 2.5% (*n* = 8) of ChR2-GFP expression was restricted to POm (top). (D) To measure VPM input, simultaneous whole-cell recordings were performed from neuronal pairs comprising an IP-derived and an OP-derived L4 neuron in acute slices, while ChR2-GFP-expressing VPM axons were stimulated with light pulses. (E) EPSP peak amplitudes for pairs of IP-derived and OP-derived neurons in response to light stimulation of VPM axons. (F) IP-derived neurons received weaker VPM input than OP-derived neurons (*n* = 18, *p* < 0.001, one-sample t test). (G) A similar arrangement was used to measure POm input. (H) EPSP peak amplitudes for pairs of IP-derived and OP-derived neurons in response to light stimulation of POm axons. (I) POm input was biased toward IP-derived neurons, which received stronger POm input than OP-derived neurons (*n* = 21, *p* = 0.002, one-sample t test). Data are represented as mean ± SEM; *n* = animals or neuron pairs. Scale bars are indicated.

While the first-order VPM represents their major input, individual L4 neurons differ in the degree to which they receive higher-order thalamic input from POm.^24–26^ One explanation for the observed relationship between embryonic progenitor type and the whisker response properties of L4 neurons is that IP-derived neurons receive greater input from higher-order thalamus. To test this directly, we used a second IUE labeling strategy to simultaneously label a population of IP-derived L4 neurons and a second population of L4 neurons that were derived from IPs in the same animal.^46,58^ To achieve this, the Tα1-Cre construct was electroporated with a dual color Cre-dependent reporter that uses the chicken β-actin (CAG) promoter to control a flexible excision cassette, whereby Cre recombination permanently switches expression from tdTomato fluorescent protein to enhanced green fluorescent protein (GFP; Figure 3A). This results in a population of GFP-labeled IP-derived L4 neurons and a separate population of TdTomato-labeled L4 neurons derived from other progenitors (OPs). “OP-derived” was therefore used to indicate neurons that are derived from progenitors in which the Tα1 promoter was not active. The OP-derived L4 population was shown to include neurons from a glutamate/aspartate transporter (Glast) positive lineage, consistent with this population including neurons derived directly from radial glial cells^32,35,39,59^ (Figure S5). Once the animals had reached P21, a thalamic injection of an adeno-associated virus (AAV) expressing ChR2-GFP was stereotaxically targeted to either VPM or POm (Figure 3A; STAR Methods). This experimental design enabled us to prepare acute brain slices at P60 and record from neuronal pairs comprising a GFP-expressing, IP-derived L4 neuron and a neighboring tdTomato-expressing, OP-derived L4 neuron while using light pulses (1 ms, 473 nm) to selectively activate ChR2-expressing thalamic axons in S1. Histological analysis of the thalamus at P60 confirmed that the majority of ChR2-GFP expression was targeted to the relevant thalamic nucleus (Figures 3B and 3C; STAR Methods).

Activation of VPM axons (Figure 3D) elicited excitatory post-synaptic potentials (EPSPs) with short onset latencies, consistent with monosynaptic inputs to both populations of L4 neurons (response delay 3.78 ± 0.33 ms in IP-derived neurons and 3.87 ± 0.40 ms in OP-derived neurons). However, the amplitude of the EPSP in the OP-derived neuron was consistently larger than in the paired IP-derived neuron (Figure 3E) so that an index capturing the relative VPM input strength to IP-derived neurons was below 0.5 (Figure 3F; 0.32 ± 0.02). Meanwhile, activation of POm axons (Figure 3G) also elicited EPSPs with short onset latencies, consistent with monosynaptic inputs to both L4 populations (response delay 3.24 ± 0.44 ms in IP-derived neurons and 3.45 ± 0.51 ms in OP-derived neurons). In contrast to VPM input, however, POm input revealed an amplitude bias in the reverse direction so that EPSPs were consistently larger in the IP-derived neuron than in the paired OP-derived neuron (Figure 3H), and an index of the relative POm input strength to IP-derived neurons was above 0.5 (Figure 3I; 0.64 ± 0.04). No systematic bias toward VPM or POm input was observed when pairs of unlabeled control L4 neurons were recorded (Figure S6). Thus, when compared to neighboring OP-derived neurons, IP-derived L4 neurons receive weaker input from VPM and stronger input from the higher-order thalamic nucleus, POm.

**Figure 4:**
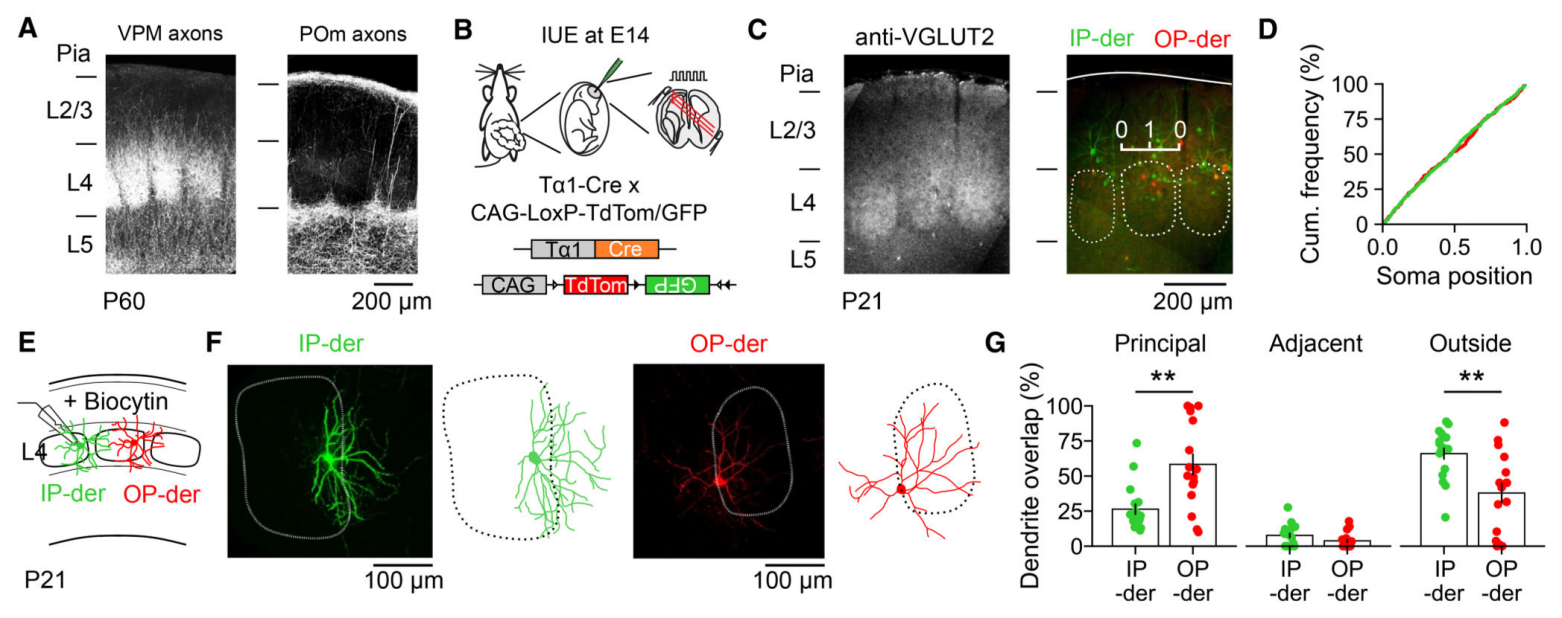
Progenitor type predicts differences in how L4 dendritic morphology relates to barrels (A) Thalamic axonal input to S1 from VPM (left) and POm (right), visualized with ChR2-GFP. (B) IUE of a Tα1-Cre and two-color Cre-dependent reporter plasmid was used to label IP-derived (green) and OP-derived (red) L4 neurons in S1. (C) VGLUT2 immunohistochemistry at P21 (left) was used to relate the distribution of labeled soma to the organization of barrels and septa. A soma position index was defined, with a value of 1 indicating a soma in the center of a barrel (right). Dashed lines indicate outlines of barrels. (D) There was no difference in the distribution of IP-derived or OP-derived L4 neurons (*n* = 537 and 351, *p* = 0.827, Mann-Whitney U test). (E) Biocytin fills were used to reconstruct the dendritic morphology of IP-derived and OP-derived neurons. (F) Example image and corresponding reconstruction of an IP-derived (left) and an OP-derived (right) L4 neuron. (G) Dendrites of OP-derived neurons were more likely to target the principal barrel than IP-derived neurons (left; *n* = 17 and 16, *p* = 0.005, Mann-Whitney U test). Dendrites of both populations exhibited a similar degree of overlap with the adjacent barrel (center; *n* = 17 and 16, *p* = 0.145, Mann-Whitney U test). Dendrites of IP-derived neurons were more likely to target the area outside of barrels, including septa (right; *n* = 17 and 16, *p* = 0.004, Mann-Whitney U test). Data are represented as mean ± SEM; *n* = neurons. Scale bars are indicated.

Thalamic input to an L4 neuron is thought to depend upon the neuron’s morphology and how this relates to the organization of barrels and septa^20,21,23^ (Figure 4A). For example, L4 neurons whose somata reside outside of a barrel can exhibit multi-whisker responses.^49^ Equally, L4 neurons can target their den-drites toward the core of a barrel, consistent with selectivity for a single PW.^41,60–62^ To investigate the soma position and dendritic morphology of IP-derived and OP-derived L4 neurons, we used the same IUE strategy at E14, and, once the animals had reached P21, we performed quantitative histology (Figure 4B). We defined a soma position index for each labeled neuron, where a value of 1 indicates a soma located at the center of a barrel, and 0 indicates the midpoint between two barrel boundaries (Figure 4C; STAR Methods). This index revealed that there was no difference in the distribution of soma positions between IP-derived and OP-derived L4 neurons (Figure 4D; mean soma position index was 0.48 ± 0.01 for IP-derived and 0.48 ± 0.02 for OP-derived). To examine dendritic morphology, we performed targeted *in vitro* whole-cell patch-clamp recordings and filled individual neurons with biocytin (Figure 4E; STAR Methods). As reported previously,^46^ there were no differences in intrinsic electrical properties between IP-derived and OP-derived L4 neurons, the frequency and amplitude of their spontaneous excitatory synaptic inputs, or in general measures of dendritic morphology (Figure S7). However, differences between the two L4 neuronal populations were observed when the reconstructions of their dendrites were co-registered to immunofluorescence images of the barrel field, and the overlap of the dendrite with the principal barrel (i.e., the barrel corresponding to the PW) was quantified (STAR Methods). The dendrites of OP-derived L4 neurons tended to target the principal barrel (Figures 4F and 4G; OP-derived overlap with principal barrel was 58.32 ± 7.78%, adjacent barrel was 3.80 ± 1.44%, and area outside barrels was 37.88 ± 7.19%). In contrast, the dendrites of IP-derived L4 neurons tended to overlap with areas outside of the principal barrel, including septa (Figures 4F and 4G; IP-derived overlap with principal barrel was 26.38 ± 4.03%, adjacent barrel was 7.67 ± 1.91%, and area outside barrels was 65.96 ± 4.31%). Therefore, while the distribution of neuronal somata was indistinguishable, the L4 neuron’s dendritic morphology was progenitor type dependent, such that the dendrites of IP-derived neurons were more likely to sample from regions outside of barrels.

### Progenitor types determine higher-order thalamic input via neuronal Lhx2 levels

**Figure 5:**
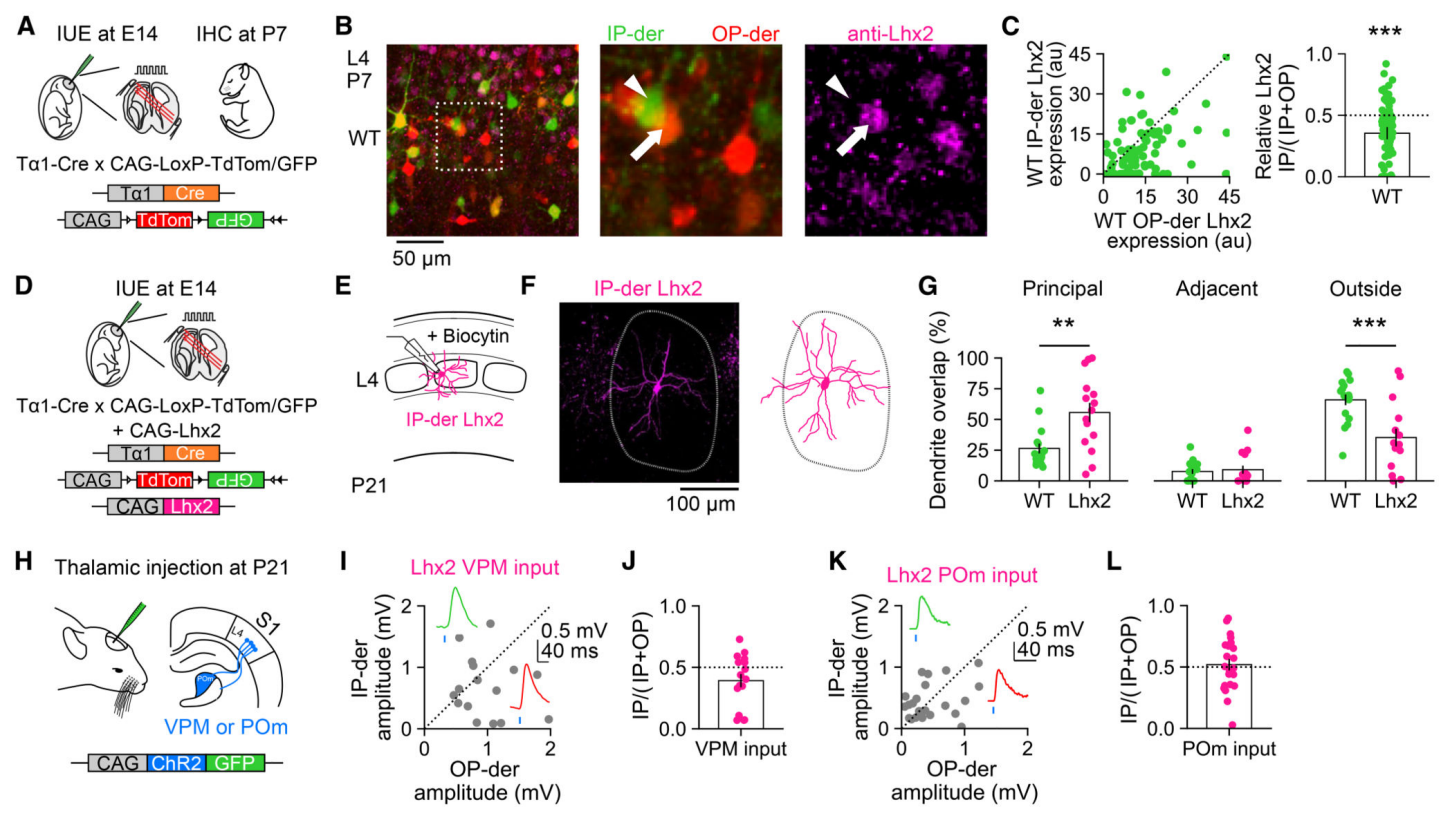
Progenitor type determines higher-order thalamic input via neuronal Lhx2 levels (A) IP-derived (green) and OP-derived (red) L4 neurons were labeled in S1, and quantitative immunohistochemistry (IHC) for Lhx2 was performed at P7. (B) IP-derived neurons expressed lower levels of Lhx2 compared to neighboring OP-derived neurons. (C) Lhx2 expression was relatively low in IP-derived neurons compared to neighboring OP-derived neurons (right; *n* = 88, *p* < 0.001, one-sample t test). (D) IUE labeling of IP-derived and OP-derived L4 neurons was combined with a CAG-*Lhx2* plasmid to increase Lhx2 expression levels. (E) Biocytin fills at P21 were used to reconstruct the dendritic morphology of IP-derived L4 neurons overexpressing Lhx2. (F) Example image and corresponding reconstruction of an IP-derived Lhx2 neuron. (G) Dendrites of IP-derived Lhx2 neurons were more likely to target the principal barrel compared to WT (left; WT data from Figure 3; *n* = 17 and 15, *p* = 0.006, Mann-Whitney U test). Dendrites of both populations exhibited a similar degree of overlap with the adjacent barrel (center; *n* = 17 and 15, *p* = 0.727, Mann-Whitney U test). Dendrites of IP-derived Lhx2 neurons were less likely to target the area outside of barrels, including septa, compared to WT (right; *n* = 17 and 15, *p* = 0.004, Mann-Whitney U test). (H) To study thalamic inputs, IUE was performed as in (D), then mice received a thalamic injection at P21 of an AAV encoding CAG-ChR2-GFP into either VPM or POm. (I) EPSP peak amplitudes for pairs of IP-derived and OP-derived Lhx2-overexpressing neurons in response to light stimulation of VPM axons. (J) No significant bias was detected in the strength of VPM input (*n* = 16, *p* = 0.06, one-sample t test). (K) EPSP peak amplitudes for pairs of IP-derived and OP-derived Lhx2 neurons in response to light stimulation of POm axons. (L) No significant bias was detected in the strength of POm input (*n* = 22, *p* = 0.687, one-sample t test). Data are represented as mean ± SEM; *n* = neurons/neuron pairs. Scale bars are indicated.

To account for this relationship between progenitor type and higher-order circuits, we reasoned that a relevant molecular mechanism would affect how the L4 neuronal progeny sample from thalamic inputs during development, and we considered whether this process involves known transcriptional programs. The transcription factor Lhx2 has been implicated previously in the post-mitotic development of neurons in S1, with an L4 neuron’s Lhx2 levels influencing its dendritic development.^63,64^ Most notably, it has been shown that reducing Lhx2 postmitotic expression in mouse S1 results in L4 neurons whose dendrites fail to properly target barrels.^64^ By combining our IUE labeling strategy with quantitative immunohistochemistry (Figure 5A), we wondered whether levels of Lhx2 protein differed between IP-derived and OP-derived neurons at P7, an age at which L4 thalamocortical circuitry is being established.^65^ Lhx2 was found to be differentially expressed in the L4 populations at P7 so that IP-derived neurons exhibited lower levels of Lhx2 than neighboring OP-derived neurons (Figures 5B and 5C; STAR Methods). This level of endogenous Lhx2 expression in IP-derived L4 neurons is consistent with previous evidence showing that low Lhx2 levels favor dendrites that do not target a specific barrel and branch into septal regions outside barrels.^64^ We therefore considered this a potential mechanism by which IPs instruct their neuronal progeny to receive higher-order thalamic input.

To test this hypothesis, animals underwent the same IUE at E14 but now combined with an Lhx2 overexpression construct, CAG-*Lhx2* (Figures 5D and S8A). This increased Lhx2 expression in IP-derived neurons (IP-derived Lhx2 neurons) when compared to IP-derived wild-type (WT) neurons at P7 and also equalized Lhx2 levels between the electroporated IP-derived and OP-derived neurons within the same tissue (Figures S8B and S8C). The mean radial soma position was comparable for Lhx2 and WT IP-derived neurons (Figures S9A and S9B), although the Lhx2 neurons exhibited more variability in their radial soma position (Figures S9C and S9D). The Lhx2-overex-pressing cells exhibited dendritic processes, consistent with a neuronal phenotype, which was confirmed in all cases by *in vitro* electrophysiological recordings. These recordings did not reveal a difference in the intrinsic electrical properties or spontaneous synaptic activity of WT and Lhx2-overexpressing IP-derived neurons (Figures S9E–S9K). Morphological reconstructions, however, revealed differences in their dendritic morphologies. In contrast to IP-derived WT L4 neurons, the dendrites of IP-derived Lhx2 L4 neurons were more targeted to the principal barrel, and their dendrites showed less overlap with areas outside barrels (Figures 5E–5G; WT overlap with principal barrel was 26.38 ± 4.03%, adjacent barrel was 7.67 ± 1.91%, and area outside barrels was 65.96 ± 4.31%; Lhx2 overlap with principal barrel was 55.63 ± 7.81%, adjacent barrel was 9.10 ± 3.25%, and area outside barrels was 47.98 ± 7.88%). These differences were not associated with overall changes to the barrel structures (Figure S10).

Furthermore, by performing ChR2-assisted mapping of thalamic inputs, we revealed that IP-derived Lhx2 L4 neurons no longer received stronger input from higher-order thalamus (Figures 5H–5L). Activation of VPM or POm ChR2-expressing axons elicited EPSPs with short onset latencies (VPM response delay was 3.85 ± 0.35 ms and 3.4 ± 0.33 ms in IP-derived and OP-derived Lhx2; POm response delay was 3.75 ± 0.47 ms and 3.88 ± 0.46 ms in IP-derived and OP-derived Lhx2, respectively).

However, unlike the WT condition (Figure 4), EPSP amplitudes were comparable between IP-derived and OP-derived Lhx2 L4 neurons, suggesting that the sampling of thalamic inputs was no longer biased under conditions of equivalent Lhx2 expression. VPM-evoked EPSP amplitudes were not different for paired IP-derived and OP-derived Lhx2 L4 neurons (Figures 5I and 5J; bias index 0.53 ± 0.04), and POm-evoked EPSP amplitudes were not different for paired IP-derived and OP-derived Lhx2 L4 neurons (Figures 5K and 5L; bias index 0.52 ± 0.05). These data indicate that low Lhx2 expression is part of the mechanism by which IP-derived WT neurons are specified to receive higher-order thalamic input and provide an experimental manipulation to test the functional significance of progenitor-dependent higher-order thalamocortical circuits *in vivo*.

### Progenitor-specified thalamocortical circuits subserve higher-order responses and sensory-evoked plasticity

**Figure 6:**
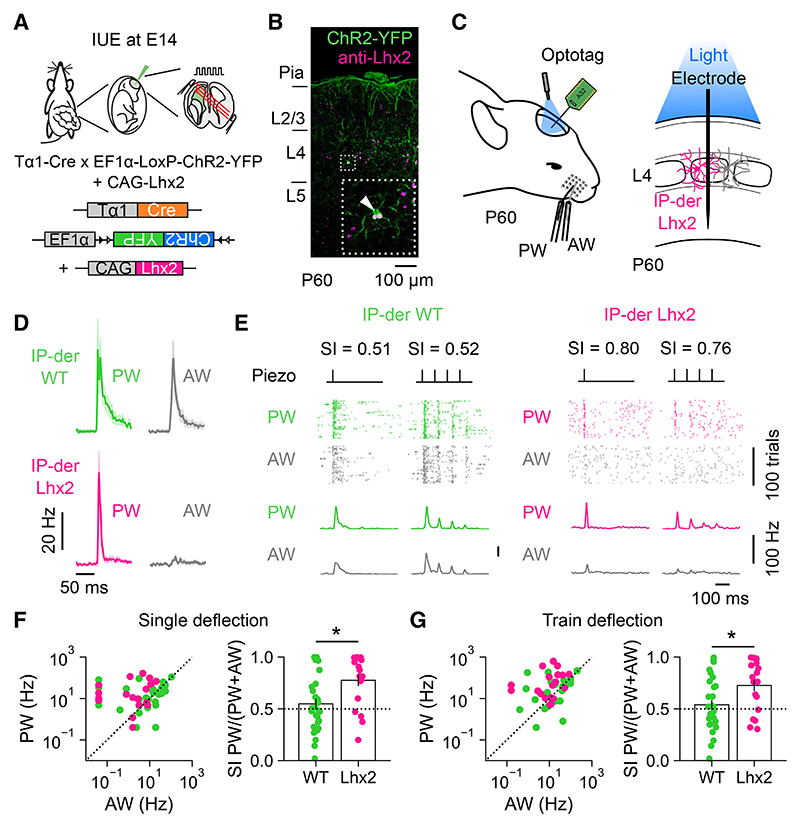
Progenitor-specified higher-order circuits contribute to multi-whisker responsivity (A) IUE of a Tα1-Cre, floxed ChR2-YFP, and CAG-*Lhx2* plasmid was used to optotag IP-derived Lhx2 L4 neurons in S1. (B) ChR2-YFP expressing IP-derived Lhx2 L4 neurons at P60. (C) The spiking activity of optotagged IP-derived Lhx2 L4 neurons was recorded in response to the deflection of the PW or AW. (D) Mean responses of IP-derived WT L4 neurons and IP-derived Lhx2 L4 neurons. (E) Spiking of an individual IP-derived WT neuron (left) and Lhx2 neuron (right) over 100 trials of either a single deflection (inner left) or trains of deflection (inner right) of the PW or AW. (F) Responses of individual IP-derived WT and Lhx2 L4 neurons to single deflection of the PW and AW (left) and the distribution of corresponding SI values (right). Compared to the WT, IP-derived Lhx2 L4 neurons showed greater selectivity for the PW and less responsivity to the AW (*n* = 29, 19; *p* = 0.011, Mann Whitney U test). (G) Responses to train deflection of the PW and AW (left) and corresponding SI values (right). Compared to WT, IP-derived Lhx2 L4 neurons showed greater selectivity for the PW and less responsivity to the AW (*n* = 29, 19; *p* = 0.014, t test). Data are represented as mean ± SEM; *n* = neurons; conventions as in Figure 2. Scale bars are indicated.

If progenitor type specifies a cortical neuron’s higher-order thalamic input, we predicted that disrupting this process would alter the cortical neuron’s sensory response properties *in vivo*. To test this, animals underwent IUE at E14 with Tα1-Cre, floxed ChR2-YFP, and CAG-*Lhx2*. This enabled individual IP-derived Lhx2 L4 neurons to be optotagged *in vivo* once the animals had reached P60 (Figures 6A–6C). PW deflection evoked robust responses in IP-derived Lhx2 L4 neurons, whereas responses to AW deflection were markedly reduced (Figures 6D and 6E). As a consequence, IP-derived Lhx2 L4 neurons exhibited higher SI values than IP-derived WT L4 neurons in response to both single and train deflections of the whiskers (Figures 6F and 6G; single deflection mean SI was 0.55 ± 0.05 for WT and 0.76 ± 0.02 for Lhx2 neurons; train deflection mean SI was 0.54 ± 0.05 for WT and 0.78 ± 0.06 for Lhx2 neurons). Thus, disrupting progenitor-specified development of higher-order thalamic input disrupts the multi-whisker response properties of IP-derived L4 neurons.

**Figure 7:**
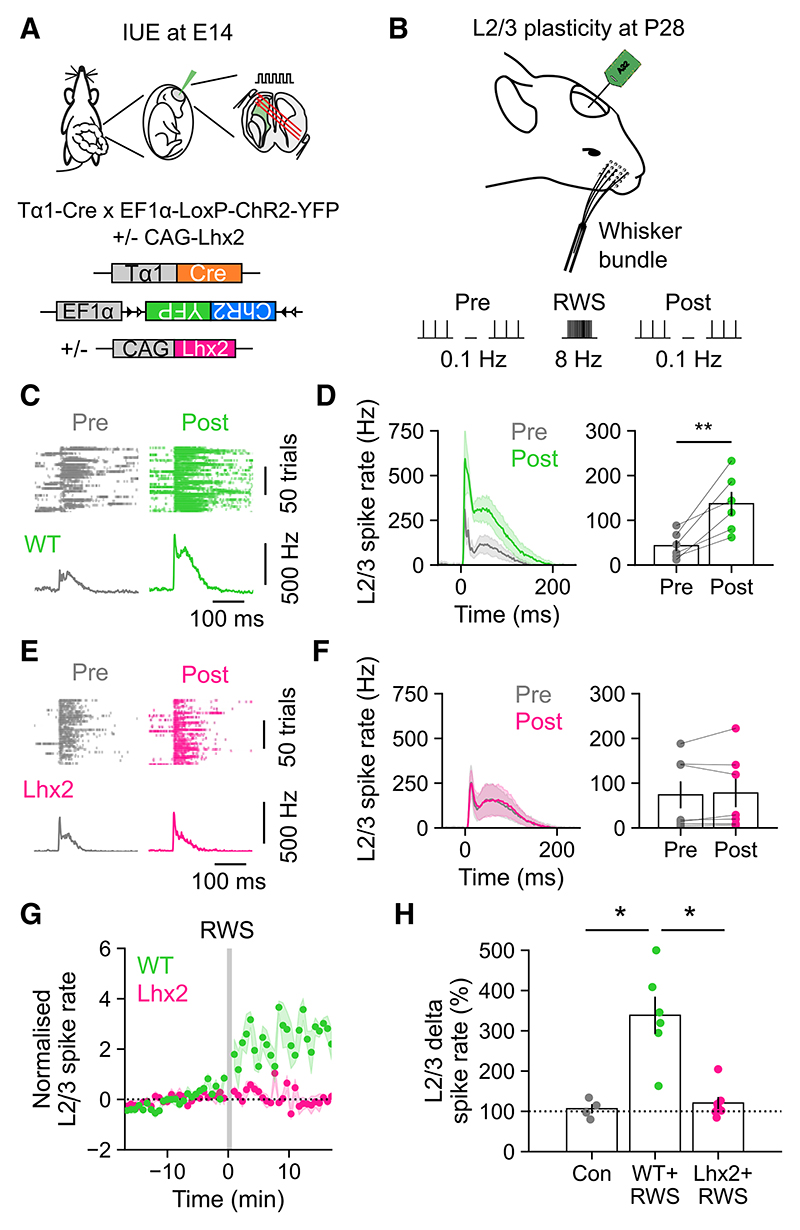
Progenitor-specified higher-order circuits support sensory-evoked plasticity (A) IP-derived WT L4 neurons underwent IUE of a Tα1-Cre and floxed ChR2-YFP plasmid. IP-derived Lhx2 L4 neurons underwent IUE of the same plasmids plus a CAG-*Lhx2* plasmid. (B) Sensory-evoked plasticity was examined in L2/3 of S1 at P28 using a rhythmic whisker stimulation (RWS) protocol (8 Hz for 60 s). The ChR2-YFP expression enabled us to use the response to a light pulse to confirm that the recording was targeting a region of S1 containing electroporated L4 neurons. (C) Raster plots and PSTHs show multi-unit L2/3 spiking activity in a WT animal. Responses to whisker deflections (0.1 Hz) are shown before (Pre) and after (Post) RWS. (D) Averaged (left) and separate (right) population data from WT animals reveal that RWS potentiated L2/3 activity (*n* = 6, *p* = 0.007, paired t test). (E) Multi-unit L2/3 activity in an Lhx2 animal. (F) Lhx2 animals did not exhibit potentiation of L2/3 activity following RWS (*n* = 7, *p* = 0.548, paired t test). (G) Normalized L2/3 multi-unit activity relative to the time of RWS (each data point is the mean of five whisker deflections, 0.1 Hz). Shading indicates SEM. (H) Delta spike rate in WT control animals that did not experience the RWS protocol (Con), WT animals that experienced RWS (WT+RWS), or Lhx2 animals that experienced RWS (Lhx2+RWS) (*n* = 4, 6, and 7; *p* = 0.001, Kruskal-Wallis test; WT control vs. WT+RWS, *p* < 0.05; WT+RWS vs. Lhx2+RWS, *p* < 0.05, WT control vs. Lhx2+RWS, *p* > 0.05, Dunn’s test). Data are represented as mean ± SEM; *n* = animals.

Higher-order thalamic inputs have been shown to promote sensory-evoked cortical plasticity through their ability to modulate the processing of first-order information.^66,67^ In rodent S1, for example, sensory-evoked plasticity between L4 and L2/3 is regulated by input from POm.^14,16^ We asked whether progenitor-specified higher-order thalamocortical circuits contribute to such forms of sensory-evoked plasticity. To test this, we compared the WT condition, under which animals underwent IUE with Tα1-Cre and floxed ChR2-YFP at E14 to target L4 excitatory neurons, with the Lhx2 condition, under which animals underwent IUE with Tα1-Cre, floxed ChR2-YFP, and CAG-*Lhx2* (Figure 7A). Once the animals had reached P28, we performed extracellular recordings of multi-unit spiking activity in L2/3 of S1, where effects of POm inputs upon sensory-evoked plasticity have been demonstrated previously^14,16^ (Figure 7B). The ChR2-YFP expression enabled us to use the response to a light pulse as online confirmation that the recordings targeted regions of S1 with electroporated L4 neurons. A rhythmic whisker stimulation (RWS) protocol was used to induce sensory-evoked long term potentiation (sLTP), as this has been shown to be effective in L2/3 of mice^14^ (Figure 7B; STAR Methods). Each experiment involved first measuring responses to multi-whisker deflections during a pre-induction (i.e., baseline) period (Pre; 0.1-Hz deflections, 100 trials), then an sLTP induction period consisting of RWS at 8 Hz for 1 min, and finally a post-induction period to measure the effects upon L2/3 responses (Post; 0.1-Hz deflection, 100 trials). In the WT condition, RWS induced robust sLTP of whisker-evoked responses in L2/3 (Figures 7C and 7D; Pre spike rate was 43.29 ± 12.14 Hz and Post spike rate was 137.06 ± 26.41 Hz), consistent with previous work.^14^ In the Lhx2 condition however, the same RWS protocol failed to elicit sLTP in L2/3 (Figures 7E–7G; Pre spike rate was 73.88 ± 30.10 Hz and Post spike rate was 78.01 ± 31.73 Hz). This effect was not associated with differences in L2/3 spiking levels during the RWS (Figure S11), and control experiments confirmed that RWS was required to induce sLTP (Figure 7H; delta spike rate in WT without RWS was 106.49 ± 11.64%, in WT with RWS was 338.59 ± 46.23%, and in Lhx2 with RWS was 120.71% ± 14.91). Similar differences in sensory-evoked plasticity in L2/3 were observed when Lhx2 overexpression was restricted to IP-derived neurons using a Cre-dependent system, supporting the idea that the effects are mediated via the IP-derived population (Figure S12). Taken together, these data are consistent with a model in which higher-order thalamic circuitry is required to elicit sensory-evoked plasticity in S1^14,16^ and demonstrate the importance of a progenitor type-dependent mechanism through which thalamic inputs are integrated in cortex.

## Discussion

Here we combined *in utero* labeling, *in vivo* electrophysiology, optical circuit mapping, and manipulation strategies to investigate how higher-order thalamocortical circuits are established. We demonstrate that, within mouse S1, the higher-order input received by a cortical neuron is related to the type of progenitor from which the neuron is derived during embryonic development. L4 excitatory neurons derived from a population of IPs were found to exhibit multi-whisker response properties and receive stronger input from the higher-order thalamic nucleus, POm. This was shown to result from progenitor-specified differences in the neuronal progeny’s molecular and dendritic development. As confirmation that these progenitor-dependent mechanisms are functionally relevant, disrupting their contribution was shown to result in cortical circuits that lacked higher-order response properties and normal sensory-evoked plasticity. This reveals an unrecognized importance for progenitor diversity in the embryonic cortex, such that neurons generated via different lineage trajectories are specified to differentially participate in thalamocortical circuits.

Consistent with previous work, S1 L4 neurons exhibited heterogeneity in their spiking responses following deflections of the PW and AW.^17,50,68,69^ By identifying L4 excitatory cortical neurons as a function of their developmental lineage, we demonstrate that this heterogeneity relates to the type of progenitor from which the neurons are derived. Previous evidence has established that clonally related excitatory neurons derived from the same individual progenitor cell can exhibit similar stimulus responsivity in the visual system.^70–72^ Our findings establish that shared sensory response properties are not only a feature of an individual clone, but can also be exhibited by cortical neurons derived from a type of progenitor. Optogenetic thalamic circuit mapping revealed that, compared to neighboring OP-derived neurons, IP-derived neurons receive greater levels of input from POm and lower levels of input from VPM, consistent with the stronger multi-whisker responses of IP-derived neurons.^4,49,51^ One could imagine also using a genetic transsynaptic labeling strategy to investigate the anatomical connectivity of the progenitor-derived L4 neurons, although a key advantage of our optogenetic circuit mapping is that it captures the strength of connectivity. Overall, our data reinforce the notion that thalamic inputs from POm to S1 show heterogeneity at a cellular level^24–26^ and highlight that progenitor type is a key determinant of how an L4 cortical neuron samples inputs from thalamus.

The emerging consensus is that all excitatory neurons in mouse cortex ultimately derive from multi-potent radial glial cells, with individual neurons being generated either directly as a result of a self-renewing asymmetric radial glial cell division or indirectly as a result of an asymmetric radial glial cell division that gives rise to a type of IP.^29,30,38–40,73^ This lineage heterogeneity creates different routes for generating excitatory neurons during an overlapping period of corticogenesis, with IPs also being considered as transit-amplifying progenitors whose function is to expand the neuronal population. Our results challenge the idea that IPs only serve to amplify neuron numbers by revealing that different lineage trajectories contribute to the diversity of thalamocortical circuitry. This extends evidence that IP populations can contribute to L4 diversity by influencing the morphology and local connectivity of their progeny^41,46^ and provides further motivation to characterize different IP subpopulations and their dynamics.^45^ A previous study used the T-box brain protein 2 (Tbr2) promoter to label a subpopulation of L4 spiny stellate neurons derived from basal IPs in mouse S1.^41^ While the functional properties of thalamic inputs were not assessed, dendritic spine density and the apposition of VGLUT2-immunopositive thalamic terminals revealed no differences compared to neighboring L4 spiny stellate neurons. It was also observed that the somata of Tbr2-derived neurons tend to be positioned closer to barrel walls and that, consistent with soma position, their dendrites show greater polarization in the horizontal axis.^41^ Given that our labeling strategy targets L4 neurons derived from a larger IP population^38–40,46,54^ and that we observe similar overall soma positions but systematic differences in thalamic input, this would suggest that IP subpopulations can differ in how they contribute to neuronal heterogeneity. More generally, our findings support the idea that neuronal diversity within excitatory neuron populations arises from heterogeneity among their embryonic progenitors. This is in line with recent evidence that the axonal patterns of long-range cortical projection neurons are related to whether the neurons derive from a radial glial cell or via an IP population,^74^ and also whether the neurons derive from molecularly distinguishable populations of radial glial cells.^75^

At a mechanistic level, our morphological studies provide a potential explanation for the neuronal progeny’s differential sampling of thalamic inputs. Reconstructions of progenitor-defined L4 neurons revealed that, while their soma positions were similar, the dendrites of individual IP-derived neurons were more likely to project outside of the principal barrel. This complements previous evidence showing that L4 neurons vary in the degree to which their dendrites target barrels^41,60–62^ and that neuronal morphology may be a general mechanism through which progenitors exert influence over their progeny.^41,74^ To further explore the underlying mechanisms, we considered whether the formation of progenitor-specified thalamocortical circuits involves known transcriptional programs, identifying Lhx2 as a factor already implicated in establishing the dendritic morphology of L4 excitatory neurons.^63,64^ The low Lhx2 levels in IP-derived L4 neurons during tha-lamocortical circuit formation is consistent with previous evidence that experimentally reducing Lhx2 levels results in dendrites that do not target barrels.^64^ To test the relevance of the low endogenous Lhx2 levels in IP-derived L4 neurons, we experimentally raised Lhx2, and this resulted in greater barrel targeting by their dendrites and an associated shift in thalamic input. Future work could extend these observations by investigating the consequences of manipulating Lhx2 in different directions within different progenitor-defined neuronal subpopulations.

Our experiments focused upon the contribution of Lhx2, and further work will be required to explore the contribution of other genes. However, Lhx2-related molecular pathways might be a fruitful direction for understanding progenitor-based heterogeneity, given that Lhx2 can be used to distinguish excitatory cortical subpopulations in L4,^74,75^ even though mature L4 neurons have generally been characterized as a molecularly homogeneous population.^46,76,77^ Indeed, it would be informative to examine the molecular pathway downstream of Lhx2, as other work has shown that Lhx2 can coordinate several activity-dependent molecular processes, including the expression of Btbd3, a transcription factor that is also required for the development of targeted dendritic morphology in L4 excitatory neurons.^64,78^ This represents an interesting point of potential convergence through which progenitor-based mechanisms could interact with activity-dependent processes during circuit formation.^79–83^

The manipulation of Lhx2 allowed us to investigate the functional importance of progenitor-specified thalamocortical circuitry in S1. Previous studies have shown that higher-order thalamocortical circuits modulate the induction of sLTP in L2/3 pyramidal neurons^14^ and that it is the co-activation of higher-order inputs and first-order inputs via L4 that forms the basis of sLTP in L2/3.^16,66,67^ In this case, POm inputs that target specific interneuron populations within the superficial layers of cortex have been shown to be important, as these mediate disinhibitory mechanisms that facilitate the potentiation of excitatory inputs from L4 to L2/3.^16^ Our experiments provide general support for such a model, by indicating that the disruption of POm inputs to IP-derived neurons within L4 is also associated with a reduced ability to induce sLTP in L2/3. Taken together, this suggests that sensory-evoked plasticity is elicited most effectively when complementary information is delivered to different neuronal compartments and/or layers—both directly to L4 and via inhibitory circuits within the superficial layers. Future work could further explore the contribution of progenitor-derived subpopulations to cortical plasticity, perhaps by asking whether IP-derived and OP-derived L4 neurons differ in their ability to directly elicit synaptic potentiation within L2/3.

In summary, we establish that an L4 cortical neuron’s receipt of lower and higher-order thalamic information relates to the progenitor type from which that neuron is derived. This progenitor specification of higher-order thalamocortical circuits provides functional evidence that progenitor types can generate distinct synaptic circuits for the differential routing of excitatory information through cortex and contribute to cortical plasticity mechanisms. It appears that the evolution of multiple progenitor types not only serves to expand cortical volume but also to generate diverse routes for the flow of subcortical information through cortex.

## Limitations of the study

Of course there are multiple limitations with our study and many remaining questions. For instance, the nature of our *in utero* labeling strategies means that, while the L4 neurons are distinguished according to their progenitor type, this is likely to be intrinsically linked to other aspects of the cells’ developmental histories. In terms of temporal processes, for example, IPs introduce an additional round of division, while radial glial cells undergo self-renewing divisions to generate neurons over a more protracted period.^35,39,47,84^ This links temporal processes to progenitor type, and a cortical progenitor’s age and lineage trajectory are associated with changes in neuronal output and fate restriction.^85–88^ Meanwhile, recent work in the hippocampus suggests that birth date can account for multiple aspects of an excitatory neuron’s connectivity and activity.^89,90^ If similar phenomena operate in cortex, then this could be one way by which progenitor types and their lineage trajectory introduce diversity to the L4 excitatory neuronal population. It would therefore be interesting to further explore the interrelationship between progenitor types, temporal processes, and thalamocortical circuitry.

## Star⋆Methods

### Key Resources Table

**Table:** 

REAGENT or RESOURCE	SOURCE	IDENTIFIER
Antibodies		
streptavidin Alexa Fluor 680	Thermo Fisher Scientific	Cat# S21378
goat anti-streptavidin	Vector Laboratories	Cat# BA-0500, RRID:AB_2336221
rabbit anti-VGlut2	Synaptic Systems	Cat# 135403, RRID:AB_887883
chicken anti-GFP	Aves Lab	Cat# GFP-1020, RRID:AB_10000240
rat anti-RFP	Chromotek	Cat# 5F8-100, RRID:AB_2336064
rabbit anti-Lhx2	Abcam	Cat# ab184337
goat anti-chicken Alexa Fluor 488	Thermo Fisher Scientific	Cat# A11039, RRID:AB_2534096
goat anti-rat Alexa Fluor 568	Thermo Fisher Scientific	Cat# A11077, RRID:AB_2534121
goat anti-rabbit Alexa Fluor 635	Thermo Fisher Scientific	Cat#A31577, RRID:AB_2536187
Bacterial and virus strains		
AAV-CAG-ChR2-GFP	UNC Vector Core, Ed Boyden	N/A
Biological samples		
Embryonic and postnatal cortices and brains from C57/Bl6 mice	This paper	N/A
Chemicals, peptides, and recombinant proteins		
4′,6-Diamidino-2-Phenylindole Dihycrochloride (DAPI)	Thermo Fisher Scientific	Cat# D1306, RRID:AB_2629482
1,1′-Dioctadecyl-3,3,3′, 3′-Tetramethylindocarbocyanine Perchlorate (DiI)	Thermo Fisher Scientific	Cat# D282
Triton X-100	Thermo Fisher Scientific	Cat# 85111
phosphate-buffered saline tablets	Thermo Fisher Scientific	Cat #003002
normal goat serum	Sigma Aldrich	Cat# G9023
paraformaldehyde	Sigma Aldrich	Cat# P6148
VectaShield	Vector Laboratories	Cat# H-1000-10
Tergazyme	Sigma Aldrich	Cat# Z273287
Urethane	Sigma Aldrich	Cat# U2500
Isoflurane	Zoetis	Cat# 42058/4195
Fast Green	Sigma Aldrich	Cat# F7252
Glycopyrronium bromide	Martindale	N/A; provided by vet
Vetergesic	Ceva Animal Health	N/A; provided by vet
Metacam	Boehringer Ingelheim	N/A; provided by vet
Marcaine	Aspen	N/A; provided by vet
EMLA cream	Aspen	N/A; provided by vet
Experimental models: Organisms/strains		
C57/Bl6 mice	Charles River	N/A
Oligonucleotides		
Flpo_XhoI_Fwr: GAGAAGCTCGAGGCCGCCACCATGGCTCCTAAGA	Sigma Aldrich	N/A
Flpe_pATerm_SacI_Rev: CTGAATGAGCTC GGGCTGCAGGTCGAGGGATCT	Sigma Aldrich	N/A
MuTbr2FwrS_Sal1: CTGCAGAAGTCGACT TTACTGAGGTGGGGTTCCAG	Sigma Aldrich	N/A
MuTbr2Rev_AgeI: TTCTGCAGACCGGTG CTTTAGCGAATCGCAGACG	Sigma Aldrich	N/A
BamHI-Flpo-Fwr: CTGCAGAAGGATTCGCC GCCACCATGGCTCCTAAGAAGAAGAGGA	Sigma Aldrich	N/A
PmeI-Flpo-Rev: ATGACGTCGTTTAAACTC AGATCCGCCTGTTGATGTAG	Sigma Aldrich	N/A
AscI-eGFPRev-Fwr: GAGAACGGCGCGCC TTACTTGTACAGCTCGTCCATGC	Sigma Aldrich	N/A
NheI-eGFPFwr-Rev: GAGACCGCTAGCG CCACCATGGTGAGCAAGG	Sigma Aldrich	N/A
Recombinant DNA		
Tα1-Cre	(Stancik et al.)^39^	N/A
Tα1-Flpo	This paper	N/A
pCAG-Flpo	(Xue et al.)^91^	http://net.addgene:60662;RRID; Addgene 60662
Tbr2-Flpo	This paper	N/A
CAG-GFP	(Zhao et al.)^92^	http://n2t.net/addgene:16664; RRID:Addgene_16664
pAAV-CAG-iCre	(Druckmann et al.)^93^	http://n2t.net/addgene:51904; RRID:Addgene_51904
Glast-Cre	(Stancik et al.)^39^	N/A
CAG-LoxP-GFP	Edward Boyden	http://n2t.net/addgene:28304; RRID:Addgene_28304
CAG-LoxP-TdTom	Edward Boyden	http://n2t.net/addgene:28306; RRID:Addgene_28306
CAG-FRT-TdTom	This paper	N/A
pAAV-CAG-fDIO-mNeonGreen	(Chan et al.)^94^	http://n2t.net/addgene:99133; RRID:Addgene_99133
pCAG-mNaChBac-T2A-tdTomato	(Xue et al.)^91^	http://n2t.net/addgene:60650; RRID:Addgene_60650
CAG-FRT-GFP	This paper	N/A
EF1α-LoxP-ChR2-YFP	Karl Deisseroth	http://n2t.net/addgene:20298; RRID:Addgene_20298
CAG-LoxP-tdTomato/GFP	(Franco et al.)^58^	N/A
CAG-Lhx2	This paper	N/A
TetO-FUW-Lhx2	(Cassady et al.)^95^	http://n2t.net/addgene:61537; RRID:Addgene_61537
CAG-LoxP-Lhx2	This paper	N/A
Software and algorithms		
MATLAB (version 2020a)	Mathworks	RRID:SCR_001622
Python (version 3.7.0)	Open source	RRID:SCR_008394 http://www.python.org
pClamp	Molecular Devices	RRID:SCR_011323
WinWCP	University of Strathclyde	RRID:SCR_014713
Zen Digital Imaging for Light Microscopy	Carl Zeiss	RRID:SCR_013672
Open Ephys	Open Ephys Production Site	https://open-ephys.org
FIJI	(Schindelin et al.)^96^	RRID:SCR_002285
ImageJ	(Schneider et al.)^97^	RRID:SCR_002285
Neurolucida 360	MBF Bioscience	RRID:SCR_016788
Kilosort	(Pachitariu et al.)^98^	RRID:SCR_016422 https://github.com/cortex-lab/Kilosort
Phy	Cyrille Rossant	https://github.com/cortex-lab/phy/
Other		
5mm platinum Tweezertrode	BTX	Cat# 45-0489
ECM 830 Pulse Generator	BTX	Cat# ECM-830
473 nm LED	LedEngin	Cat# 905-3870
DC temperature regulation system	FHC inc	Cat# 40-90-8D
Pneumatic dental drill	Foredom	Cat# H.MH-170
32-channel electrode	Neuronexus	Cat# A32 - Rev 3.3
Mouse stereotaxic frame	Stoelting	Cat# 51730
Mouse stereotaxic frame	Kopf	Cat# 963
Vibrating microtome	Microm	Cat# HMV650V
Aquisition board	OpenEphys	N/A
PulsePal	OpenEphys	N/A
Piezoelectric bending actuator	Piezo Technics	N/A
Piezo controller	Piezo Technics	N/A
RHD2132 amplifier	Intan	Cat# RHD2132
NPD36 connector	Omnetics	Cat# NPD36
Vicryl	Ethicon	W9500T
Prolene	Ethicon	W8890
Injection micropipettes	Blaubrand intraMARK	Cat# BR708707-1000EA
1.5 mm outer diameter borosilicate capillaries	Warner Instruments	Cat# 64-0793
1.2 mm outer diameter borosilicate capillaries	Warner Instruments	Cat# 64-0790

### Resource Availability

#### Lead contact

- Further information and requests for resources should be directed to and will be fulfilled by the lead contact, Colin Akerman (colin.akerman@pharm.ox.ac.uk).

#### Materials availability

- New reagents generated in this paper are available from the lead contact upon reasonable request.

### Experimental Model and Study Participant Details

Experiments were performed using C57/BL6 wildtype mice, which were bred, housed, and used in accordance with the United Kingdom Animal Scientific Procedures Act 1986 under personal and project licences granted by the United Kingdom Home Office. Ethical approval for the animal experimentation was granted by the Animal Welfare and Ethical Review Body at the University of Oxford. Animals of both sexes were used throughout the study and were randomly assigned to the experimental groups, such that sex was not considered an influencing factor. Breeding females were used for *in utero* electroporation and were checked daily for plugs, with the day of plugging being considered embryonic day (E) 0.5. The ages of animals is stated for each experiment in the Results.

### Method Details

#### In utero electroporation

*In utero* electroporation (IUE) was performed using standard procedures at E14 consistent with previous studies targeting S1 L4^52,53^. Briefly, pregnant females were anesthetized using isoflurane (Zoetis). Buprenorphine (Vetergesic; 0.1 mg/kg) and meloxicam (Metacam; 5 mg/kg) were administered subcutaneously. The uterine horns were exposed by midline laparotomy. A mixture of plasmid DNA (~2 μg/μL) and 0.03% fast green dye (Sigma Aldrich) was injected intraventricularly using micropipettes pulled from borosilicate glass capillaries (1.5 mm outer diameter, Warner Instruments), through the uterine wall and amniotic sac. The different plasmid DNA were as follows:

(i) “Tα1-Cre”, in which Cre recombinase is under the control of a portion of the Tubulin alpha-1 (Tα1) promoter and was a gift from Tarik Haydar.^39^
(ii) “Tα1-Flpo”, in which codon optimized Flp recombinase is under the control of a portion of the Tα1 promoter. To generate Tα1-Flpo, Cre recombinase and its associated 3' poly(A) sequences were removed from Tα1-Cre using XhoI/SacI and replaced with Flpo recombinase and its associated poly(A) sequences from pCAG-Flpo (Addgene plasmid # 60662; http://net.addgene: 60662; RRID; Addgene 60662; a gift from Massimo Scanziani^91^). The Flpo sequence was amplified using Phusion DNA poly-merase (New England Biolabs) and the following primers:
  Flpo_XhoI_Fwr: GAGAAGCTCGAGGCCGCCACCATGGCTCCTAAGA.
  Flpe_pATerm_SacI_Rev: CTGAATGAGCTCGGGCTGCAGGTCGAGGGATCT.
(iii) “Tbr2-Flpo”, in which Flp recombinase is under the control of a portion of the T-box brain protein 2 (Tbr2) promoter. To generate Tbr2-Flpo, first Tbr2-GFP was generated in the retroviral backbone CAG-GFP (Addgene plasmid # 16664; http://n2t.net/addgene:16664; RRID:Addgene_16664; a gift from Fred Gage^92^). The CAG promoter was removed using restriction enzymes SalI/AgeI and replaced with a 2.5kb fragment of the mouse Tbr2 promoter based on previous work.^54^ The promoter region was amplified from mouse brain genomic DNA using the following primers:
  MuTbr2FwrS_Sal1: CTGCAGAAGTCGACTTTACTGAGGTGGGGTTCCAG.
  MuTbr2Rev_AgeI: TTCTGCAGACCGGTGCTTTAGCGAATCGCAGACG.
  The GFP sequence was subsequently removed and replaced with a small multiple cloning site (MCS) containing sites AgeI, HindIII, StuI and BamHI inserted between the Tbr2 promoter and the improved Cre recombinase sequence (iCre) to generate Tbr2-Cre. iCre was amplified from pAAV-CAG-iCre (Addgene plasmid # 51904; http://n2t.net/addgene:51904; RRID:Addgene_51904; a gift from Jinhyun Kim^93^). Subsequently, Tbr2-Flpo was generated by removing the iCre sequence using a BamHI/PmeI digest and replacing it with the Flpo sequence amplified from pCAG-Flpo (Addgene plasmid # 60662; http://n2t.net/addgene:60662; RRID: Addgene_60662; a gift from Massimo Scanziani) using the following primers:
  BamHI-Flpo-Fwr: CTGCAGAAGGATTCGCCGCCACCATGGCTCCTAAGAAGAAGAGGA
  PmeI-Flpo-Rev: ATGACGTCGTTTAAACTCAGATCCGCCTGTTGATGTAG.
(iv) “Glast-Cre”, in which Cre recombinase is under the control of a portion of the glial high affinity glutamate/aspartate transporter (Glast) promoter and was a gift from Tarik Haydar.^39^
(v) “CAG-LoxP-GFP”, a single color Cre-dependent reporter that uses the chicken β-actin (CAG) promoter to control a flexible excision cassette, whereby Cre recombination permanently turns on expression of enhanced GFP. CAG-LoxP-GFP was a gift from Edward Boyden (Addgene plasmid # 28304; http://n2t.net/addgene:28304; RRID:Addgene_28304).
(vi) “CAG-LoxP-TdTom”, a single color Cre-dependent reporter that uses the chicken β-actin (CAG) promoter to control a flexible excision cassette, whereby Cre recombination permanently turns on expression of TdTomato. CAG-LoxP-TdTom was a gift from Edward Boyden (Addgene plasmid # 28306; http://n2t.net/addgene:28306; RRID:Addgene_28306).
(vii) “CAG-FRT-TdTom”, a single color Flpo-dependent reporter that uses the CAG promoter to control a flp-dependent double-floxed inverted open reading frame, whereby Flpo expression permanently switches on the expression of tdtomato. CAG-FRT-TdTom was derived from pAAV-CAG-fDIO-mNeonGreen (Addgene plasmid # 99133; http://n2t.net/addgene:99133; RRID:Addgene_99133; a gift from Viviana Gradinaru^94^). The mNeonGreen sequence was removed using a AscI/NheI digest and replaced with a Tandem Tomato (TdTomato) sequence that was amplified from pCAG-mNaChBac-T2A-tdTomato (Addgene plasmid # 60650; http://n2t.net/addgene:60650; RRID:Addgene_60650; a gift from Massimo Scanziani^91^) using the following primers to amplify both fluorescent protein sequences:
  AscI-eGFPRev-Fwr: GAGAACGGCGCGCCTTACTTGTACAGCTCGTCCATGC.
  NheI-eGFPFwr-Rev: GAGACCGCTAGCGCCACCATGGTGAGCAAGG.
(viii) “CAG-FRT-GFP”, a single-color Flpo-dependent reporter that uses the CAG promoter to control a flp-dependent double-floxed inverted open reading frame, whereby Flpo expression permanently switches on the expression of GFP. CAG-FRT-GFP was derived from pAAV-CAG-fDIO-mNeonGreen by replacing the mNeonGreen with GFP from CAG-GFP.
(ix) “EF1α-LoxP-ChR2-YFP” (pAAV-EF1a-double floxed-hChR2(H134R)-EYFP-WPRE-HGHpA; Addgene #20298; http://n2t.net/addgene:20298; RRID:Addgene_20298; a gift from Karl Deisseroth), in which Cre recombination turns on the expression of channelrhodopsin-2 fused to enhanced yellow fluorescent protein (ChR2-YFP) under the control of the human elongation factor-1a promoter.
(x) “CAG-LoxP-tdTomato/GFP”, which uses the chicken β-actin (CAG) promoter to control a flexible excision cassette, whereby Cre recombination permanently switches expression from tdTomato to GFP, and which was a gift from Ulrich Müller.^58^
(xi) “CAG-Lhx2”, in which mouse Lhx2 is under the control of the CAG promoter. To generate CAG-Lhx2, mouse Lhx2 cDNA was amplified from TetO-FUW-Lhx2 (Addgene #61537; http://n2t.net/addgene:61537; RRID:Addgene_61537; a gift from Rudolf Jaenisch^95^) and cloned into the AAV backbone derived from pAAV-CAG-iCre (Addgene #51904; http://n2t.net/addgene:51904; RRID:Addgene_51904; a gift from Jinhyun Kim).
(xii) “CAG-LoxP-Lhx2”, in which Cre recombination turns on the expression of mouse Lhx2 under the control of the CAG promoter. To generate CAG-LoxP-Lhx2, mouse Lhx2 cDNA was amplified from TetO-FUW-Lhx2 (Addgene #61537; http://n2t.net/addgene:61537; RRID:Addgene_61537; a gift from Rudolf Jaenisch) and cloned into the backbone derived from CAG-LoxP-GFP.

Plasmids were injected as a 1:1 ratio and the total volume injected per embryo was ~2 μL. The anode of a 5 mm Platinum Twee-zertrode (BTX) was placed over the dorsal telencephalon outside the uterine muscle. Five pulses (50 ms duration separated by 950 ms) at 36 V were delivered with an ECM 830 pulse generator (BTX). The uterine horns were placed back inside the abdomen, the cavity filled with warm physiological saline, and the abdominal muscle and skin incisions were closed with Vicryl (Ethicon) and Prolene (Ethicon) sutures, respectively. Dams were monitored until the birth of the pups and further analgesia was provided, as appropriate.

#### Postnatal intrathalamic viral injections

Animals that had undergone IUE were used for targeted intrathalamic injections at P21. Briefly, mice were anesthetized using iso-flurane and placed in a stereotaxic frame (Kopf Instruments). Vetergesic (0.1 mg/kg) was administered subcutaneously, and EMLA cream (Aspen) was applied to the scalp. An incision was made to expose the skull. Bregma and lamda were located and a small craniotomy was performed to expose the neocortex. Injections were targeted to either the ventral posteromedial nucleus (VPM; 1.8 mm lateral to bregma, 1.4 mm posterior; 3.1 mm deep from pia), or the posterior medial nucleus (POm; 1.4 mm lateral to bregma, 2.1 mm posterior; 3 mm deep from pia) of the thalamus. 120–240 nL of an adeno-associated virus (AAV) carrying CAG-ChR2-GFP, in which ChR2-GFP was under the control of the CAG promoter (Boyden, UNC Vector Core), was injected over a period of 8 min using a pulled glass micropipette (Blaubrand intraMARK). The craniotomy was covered, and the skin closed with Vicryl sutures. Further analgesia was provided, as appropriate.

#### *In vitro* slice preparation and recordings

Acute slices were prepared from postnatal animals from P21 (range P21 – 28), or from P60 (range P60 – 75) where a postnatal intra-cerebral injection had been performed. Animals were anesthetized using isoflurane and decapitated. Thalamocortical 350–400 μm slices (55° with respect to midline) were cut using a vibrating microtome (Microm). Slices were prepared in artificial cerebrospinal fluid (aCSF) containing (in mM): 65 sucrose, 85 NaCl, 2.5 KCl, 1.25 NaH_2_PO_4_, 7 MgCl_2_, 0.5 CaCl_2_, 25 NaHCO_3_ and 10 glucose, pH 7.2–7.4, bubbled with carbogen gas (95% O_2_/5% CO_2_). Slices were immediately transferred to a storage chamber containing aCSF (in mM): 130 NaCl, 3.5 KCl, 1.2 NaH_2_PO_4_, 2 MgCl_2_, 2 CaCl_2_, 24 NaHCO_3_ and 10 glucose, pH 7.2–7.4, at 32°C, and bubbled with carbogen gas. When required, slices were transferred to a recording chamber and continuously superfused with aCSF bubbled with carbogen gas with the same composition as the storage solution (32°C and perfusion speed of 2 mL/min). Whole-cell current-clamp recordings were performed using glass pipettes, pulled from borosilicate glass capillaries (1.2 mm outer diameter, Warner Instruments), containing (in mM): 110 potassium gluconate, 40 HEPES, 2 ATP-Mg, 0.3 Na-GTP, 4 NaCl and 4 mg/mL biocytin (pH 7.2–7.3; osmolarity 290–300 mOsmol/L).

#### *In vitro* stimulation, recording, and analysis

Recordings were made using a Multiclamp 700B (Molecular Devices) amplifier and acquired using WinWCP (University of Strath-clyde, UK) or pClamp (Molecular Devices) software. All recordings were low pass filtered at 2 kHz and digitized at a sampling frequency of 10 kHz. Slices were placed into a recording chamber and barrels were visualized in layer 4 (L4) under brightfield illumination. The distribution of cells labeled by IUE meant that the intrinsic electrophysiological properties and morphology of neurons were sampled across the extent of S1. Single L4 excitatory neurons within barrels were identified and targeted using video assisted Dodt contrast imaging. Progenitor identity was confirmed using fluorescent light. The intrinsic properties of the recorded neurons were assessed using a variety of protocols consisting of hyperpolarizing and depolarizing current steps (from −300 to +600 pA, 100 Pa steps) in current clamp. Measurements included resting membrane potential, spike threshold, spike frequency, spike amplitude and inter-spike interval. The resting membrane potential was calculated from a pre-stimulus period of 0 pA current injection, averaged over 10 sweeps. The values of spike threshold voltage were calculated manually from the recorded traces. All other measures were calculated using Python. Spontaneous excitatory postsynaptic currents (sEPSCs) were recorded in voltage clamp whilst holding the cell at −70 mV. Each sEPSC recording was 10 min long. The pClamp event detection tool was used to create a standardized template by manually selecting ~200 spontaneous events. This template was then used to automatically detect sEPSCs. Monosynaptic thalamic inputs to L4 neurons were studied by stimulating ChR2-GFP expressing axons in L4, which originated from either POm or VPM. Photoactivation of ChR2 was achieved using 1 ms light pulses via an LED (473 nm; 3.8–21.6 mW/mm^2^, LedEngin) and the amplitude of short-latency, time-locked, light-evoked excitatory postsynaptic potentials (EPSPs) were measured from pairs of simultaneously recorded L4 neurons from the average of 10–40 sweeps. Light intensity was adjusted to produce low amplitude monosynaptic EPSPs (mean peak <3 mV), to minimize the chance of recruiting polysynaptic activity.

#### *In vivo* recording conditions

Extracellular recordings were performed from P60 (range P60 – 75) for selectivity experiments, and from P28 (range P28 – 35) for plasticity experiments. Animals were anesthetized with 25% urethane (1 g/kg; Sigma) in phosphate-buffered saline (PBS; Thermo Fisher), then mounted in a stereotaxic frame (Stoelting) and continuously supplied with oxygen (0.3 mL/min) throughout the recording. Glycopyrronium bromide (Glycopyrrolate; 0.01 mg/kg) was administered subcutaneously, and Marcaine (Aspen) was applied to the scalp. A heat mat controlled by a direct current temperature regulation system (FHC inc.) was used to maintain body temperature at 37°C. A single incision was made to remove the skin from the skull and a craniotomy of ~2 mm diameter was performed using a pneumatic dental drill (Foredom). A 32-channel single-shank electrode (Neuronexus) was repeatedly sub-merged in 1,1'-Dioctadecyl-3,3,3',3'-Tetramethylindocarbocyanine Perchlorate (DiI) lipophilic dye (2.5 mg/mL, in 70% ethanol, Thermo Fisher) and then slowly inserted into the cortex at a 20° angle from the vertical (with respect to bregma: 3 mm lateral,1.2 mm posterior; 0.9 mm deep from pia).

#### *In vivo* stimulation and recording protocols

The electrode was connected to an acquisition board (OpenEphys) using a NPD36 connector (Omnetics) and a RHD2132 amplifier (Intan). In order to deflect two single whiskers independently, borosilicate capillaries (1.5 mm outer diameter, Warner Instruments) were attached to two piezoelectric bending actuators (Piezo Technics). Deflection was achieved using a single 100 Hz sinusoidal waveform controlled by a Piezo Controller (Piezo Technics). Photoactivation of ChR2 was achieved using 10 ms light pulses via an LED (473 nm, 45 mW/mm^2^, LedEngin) positioned above the cortical surface. All stimulus protocols were generated in MATLAB and delivered via a PulsePal pulse train generator (OpenEphys). The principal whisker (PW) relative to the electrode insertion site was manually identified online by the presence of a robust, short-latency spiking response following whisker deflection and a characteristic current source density profile, calculated as the second spatial derivative of the local field potential (LFP). The adjacent whisker (AW) was defined as the whisker immediately rostral to the principal whisker. If this whisker was missing or did not evoke a response, the whisker immediately caudal was used. The identities of the PW and AW were confirmed offline using the population spike rate and response latency of all L4 excitatory neurons from a given animal.

To study sensory long-term potentiation (sLTP), we performed a rhythmic whisker stimulation (RWS) protocol as has been described previously.^14^ Before (“pre”) and after (“post”) RWS, responses were measured by deflecting multiple whiskers (~10) with a single piezoelectric bending actuator at 0.1 Hz for 100 trials. During RWS, the whiskers were deflected for 1 min at 8 Hz (i.e., 100 Hz waveform every 125 ms). In all experiments, baseline activity was recorded for between 30 min and 1 h following electrode insertion. Electrodes were immersed in 1% Tergazyme (Sigma Aldrich) for 2 h between recording sessions. Data was acquired at 30 kHz. To obtain multiunit activity, data was bandpass filtered between 300 and 6000 Hz. Multiunit activity was detected using the median absolute deviation of the filtered signal.^99^ To obtain the LFP, data was lowpass filtered under 300 Hz and a 50 Hz notch filter was applied. To obtain single unit activity, data was spike sorted using Kilosort,^98^ and curated in Phy.^100^

#### *In vivo* analysis

Cortical layers were defined using the PW current source density. The shortest latency current source density sink evoked upon stimulation of the principal whisker was defined as L4. Electrode channels above L4 were assigned as L2/3. Regular spiking (primarily excitatory) neurons were separated from fast-spiking (putative interneurons) neurons using a spike waveform trough-to-peak time of 0.5 ms as the separation criterion.^101^ To identify neurons that were directly activated by ChR2, repeated light pulses were delivered to the probe insertion site. Optotagged neurons were defined by a mean response latency <5 ms. To control for the recruitment of polysynaptic light-evoked activity, the AMPA receptor blocker DNQX (10 mM, Tocris) was applied to the cortical surface in a subset of experiments. The stimulus-evoked response window for single whisker deflection was defined as 50 ms. In all experiments, spontaneous activity was calculated on a trial-by-trial basis as the average spike rate of a given unit or channel and subtracted from stimulus-evoked responses. Response latency was calculated as the 1 ms time bin containing the first spike 5–20 ms following whisker deflection in each trial. The selectivity index for individual neurons in response to single deflection was calculated as: RPW/RPW + RAW, where RPW and RAW were the cumulative spontaneous subtracted spike count in the 50 ms following deflection of the PW or AW, respectively (i.e., Figure 1). For train deflection RPW and RAW were the total sum of the spike counts in four separate 50 ms windows following each deflection. The stimulus-evoked response window for the deflection of multiple whiskers (i.e., Figure 7) was defined as 250 ms.

#### Histological analysis

To confirm the location of the postnatal intrathalamic viral injections, brains were fixed by cardiac perfusion of PBS followed by 4% paraformaldehyde (PFA, Sigma Aldrich), stored in 4% PFA for an additional 24 h, after which they were washed and stored in PBS. 50 μm thick brain sections were prepared using a vibrating microtome (Microm), counter-stained with 4',6-Diamidino-2-Phenylindole, Dihydrochloride (DAPI, 1:10000, Thermo Fisher) and mounted on slides with VectaShield (Vectorlabs). Fluorescent images were acquired with an LSM 880 confocal microscope (Zeiss) equipped with 488 nm, 561 nm, and 633 nm lasers and a 20x water-immersion objective (W Plan-Apochromat) using ZEN software (Zeiss). Sections that contained thalamus were closely examined, overlaid with the corresponding section from the mouse brain atlas,^102^ and landmarks were used to delineate the VPM and POm as ROIs. Using thresholding scripts in FIJI^96^ (ImageJ^97^), the relative proportion of ChR2-YFP expression that fell within the ROI was calculated across 3 sections per brain.

For whole-brain histology, 50 μm brain sections were prepared as above. For morphological reconstructions of individual neurons following whole-cell patch-clamp recordings, acute brain slices were fixed in 4% paraformaldehyde (PFA, Sigma Aldrich) in 0.1 M PBS (pH 7.4). Biocytin-filled cells were visualized using repeated incubation with streptavidin Alexa Fluor 680 (1:1000, Thermo Fisher), boosted using anti-streptavidin (1:1000, goat, Vector Laboratories). The standard immunohistochemistry protocol was as follows. Sections were washed three times in PBS for 5 min, then blocked in 20% normal goat serum (NGS, Sigma Aldrich) in 0.1% Triton X-(Thermo Fisher) in PBS (PBST) for 2 h at RT. Sections were washed in PBS and incubated overnight with primary anti-body diluted in 0.1% PBST at 4°C. Primary antibodies included: anti-VGlut2 (1:250, rabbit, Synaptic Systems), anti-GFP (1:1000, chicken, Aves Lab), anti-RFP (1:1000, rat, Chromotek), and anti-Lhx2 (1:500, rabbit, Abcam). VGLUT2 staining was facilitated using heated antigen retrieval at 92°C in 10 mM fresh sodium citrate (pH 6.0) for 30 min prior to primary antibody incubation. Slices were washed in PBS and were incubated for 2 h with secondary antibodies diluted in 0.1% PBST at RT. Secondary antibodies included: anti-chicken Alexa Fluor 488 (1:1000, goat, Thermo Fisher), anti-rat Alexa Fluor 568 (1:1000, goat; Thermo Fisher), and anti-rabbit Alexa Fluor 635 (1:1000, goat, Thermo Fisher). Sections were counter-stained and slide-mounted, as above. All cell counting, localization and fluorescence analysis was performed in FIJI (ImageJ).

Soma coordinates were labeled manually, and a reference line drawn along the L4/L5 boundary based on VGLUT2 fluorescence. The image and soma coordinates were then transformed with respect to the reference line, thereby straightening the cortical layers whilst maintaining relative soma position. Barrel boundaries were detected automatically based on VGLUT2 fluorescence in L4. All soma were assigned a barrel index score with 1 indicating a soma located in the middle of a barrel, and 0 indicating a soma located at the midpoint between two barrel boundaries.

Biocytin-filled L4 spiny stellate neurons in S1 were reconstructed using Neurolucida and Neuroexplorer software (MBF Bioscience) and co-registered to immunofluorescence images of the barrel field. The identity of the principal barrel was defined anatomically as the barrel with which the majority of dendrites overlapped. The adjacent barrel was defined as the next closest barrel. Dendritic overlap was quantified as a percentage of total dendritic length. Neurons with <5% total dendritic length within any barrel were excluded from further analyses. Lhx2 expression levels were quantified as a ratio of the mean pixel intensity (MPI) of the cell body of an IP-derived L4 neuron over the mean MPI of three neighboring OP-derived L4 neurons within the same z-plane and within a 100 3 100 μm region of interest. For barrel analysis, barrel morphology was visualised using a combination of DAPI and brightfield fluorescence. Barrel outlines were drawn and analyzed in FIJI (ImageJ).

### Quantification And Statistical Analysis

All data are presented as mean ± standard error unless otherwise stated. All statistical analysis was performed in Python using SciPy or in GraphPad. Continuous data were assessed for normality and appropriate parametric or non-parametric statistical tests were applied. Details of the specific statistical tests can be found within the relevant figure legends (*p < 0.05, **p < 0.01, ***p < 0.001). Sample sizes were based on detecting Cohen’s D effect sizes of approximately 1 (range 0.8–1.2) and therefore smaller effects sizes could be missed, as the probability of type II errors increase as effect sizes decrease. The source data contributing to the main figures and supplemental figures is provided as a separate document (Table S1).

## Supplementary Material

Supplemental information can be found online at https://doi.org/10.1016/j.celrep.2024.114157.

Supplementary Information

## Data Availability

The raw data contributing to the main figures and supplemental figures is provided as a separate document (Table S1). The datasets presented in this paper are available from the lead contact upon reasonable request. This paper does not report original code. Any additional information required to reanalyse the data reported in this paper is available from the lead contact upon reasonable request.

## In brief

Buchan et al. demonstrate that the thalamic input received by a layer 4 excitatory cortical neuron in mice is influenced by the neuron’s embryonic progenitor type. Neurons derived from intermediate progenitors receive greater input from higher-order thalamus, which reflects their dendritic morphology and molecular mechanisms specified by the progenitors.

## Highlights

- Progenitor type determines an excitatory cortical neuron’s input from thalamus
- L4 neurons derived from intermediate progenitors receive more higher-order thalamic input
- This reflects progenitor-specified effects upon fine-scale dendritic development
- Progenitor-specified circuits dictate higher-order sensory responses and plasticity
