# Supplementary material for: Higher-order thalamocortical circuits are specified by embryonic cortical progenitor types in the mouse brain
Source: Cell Rep. Author manuscript; Available in PMC 2025 Jul 29. (PMC7617957; doi:10.1016/j.celrep.2024.114157)
Supplement: Supplementary Information [file EMS206925-supplement-Supplementary_Information.zip › 1-s2.0-S2211124724004856-mmc1.pdf]

Cell Reports, Volume *43*

Supplemental information

Higher-order thalamocortical circuits are specified by embryonic cortical progenitor types in the mouse brain

Matthew J. Buchan, Gemma Gothard, Kashif Mahfooz, Joram J. van Rheede, Sophie V. Avery, Alexios Vourvoukelis, Alexander Demby, Tommas J. Ellender, Sarah E. Newey, and Colin J. Akerman

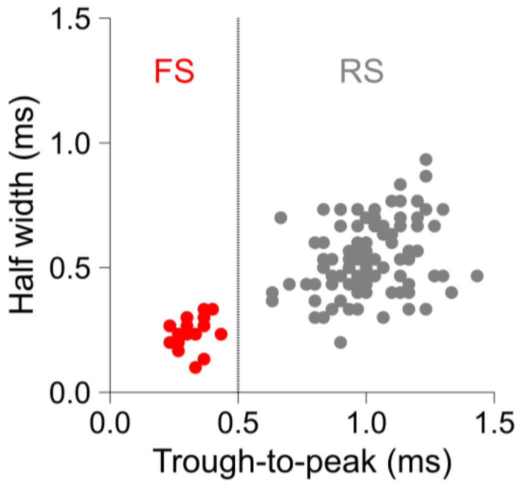

Supplementary Figure 1: *In vivo* identification of putative excitatory neurons from extracted waveform properties. Related to Figure 1. Regular-spiking (RS; primarily excitatory) neurons were distinguished from fast-spiking (FS; primarily interneurons) neurons based on the waveform properties of single units isolated using Kilosort. A trough-to-peak time of 0.5 ms was used as the separation criterion (FS trough-to-peak was 0.31 ± 0.01 ms and RS trough-to-peak was 1.01 ± 0.02 ms, n = 21 and 100, respectively), in keeping with previous studies in rodent cortex [S1]. Data represented as mean ± SEM, n = neurons.

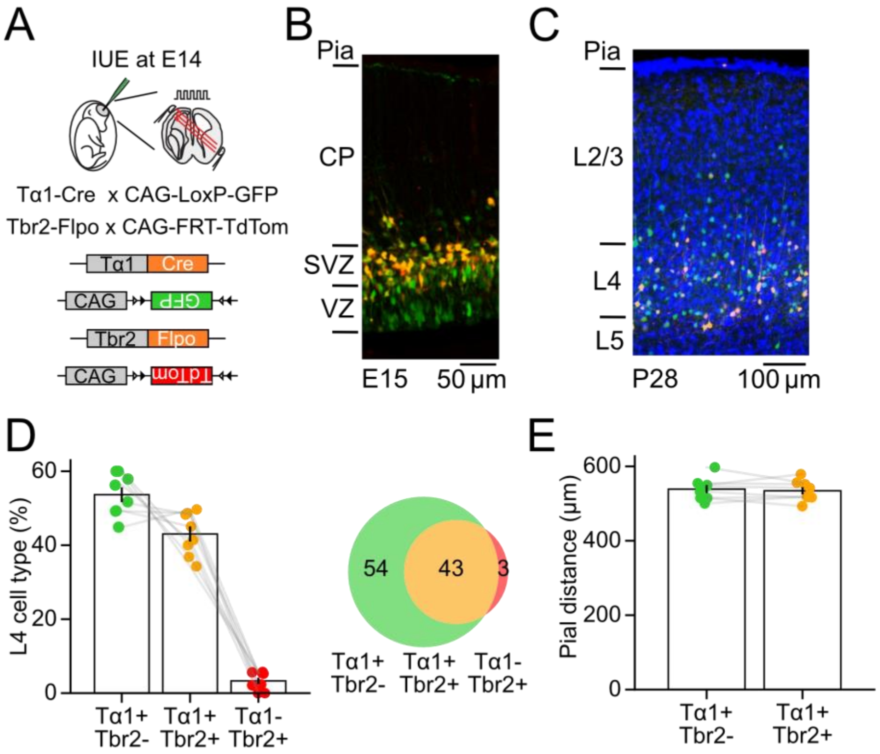

Supplementary Figure 2: *In utero* electroporation with Tα1-Cre labels IPs that contribute to the L4 neuron population. Related to Figure 2. (**A**) In order to label IPs and their progeny, E14 animals underwent IUE of four plasmids encoding Tα1-Cre, Cre-dependent GFP, Tbr2-Flpo, and Flpo-dependent TdTomato. Previous work at these ages has shown that the Tbr2 promoter can be used to label a subpopulation of IPs within the SVZ, referred to as basal IPs [S2-S4]. (**B**) 24 hours following IUE at E14, fluorescently labelled progenitors were observed in the VZ and SVZ. In all embryos examined (n = 6), the Tbr2-positive cells (i.e. expressing TdTomato) exhibited multipolar morphologies and were largely restricted to the SVZ, where basal IPs are known to reside [S4-S5]. Almost all Tbr2-positive cells were also Tα1-positive (i.e. expressing GFP), in agreement with evidence that Tα1 is expressed by basal IPs [S7], and suggesting that Tα1 labelling encompasses basal IPs. A second population of cells were Tα1-positive and Tbr2-negative (i.e. expressing GFP but not TdTomato). These cells had short radial morphologies and resided in the VZ, where some could be seen dividing at the ventricular wall, consistent with an apical IP identity [S8-S9]. (**C**) To map the progeny of these progenitor populations, L4 neurons were studied at P28 following the IUE shown in 'A'. (**D**) The majority of labelled L4 neurons at P28 were derived from a Tα1-positive/Tbr2-negative lineage (53.66 ± 2.00 %) and most of the remaining labelled neurons derived from a Tα1-positive/Tbr2-positive lineage (43.03 ± 2.05 %). Extremely few neurons were derived from a Tα1-negative/Tbr2-positive lineage (3.31 ± 0.88 %), again consistent with the idea that Tα1 labelling encompasses basal IPs. (**E**) Neurons derived from the Tα1-positive/Tbr2-negative lineage and Tα1-positive/Tbr2-positive lineage were located at comparable distances from the pia (Tα1-positive/Tbr2-negative was 538.78 ± 10.80 µm, Tα1-positive/Tbr2-positive was 534.30 ± 9.80 µm, n = 8, p = 0.59, paired t-test). This suggests the two populations are born over a similar period of embryonic development, consistent with the idea that both derive from transiently dividing intermediate progenitors, rather than one population including progenitors that undergo self-renewing divisions [S9]. Overall, these results support the conclusion that the Tα1 labelling strategy targets a population of IPs that comprise both apical and basal IPs. Data represented as mean ± SEM, n = animals. Scale bars are indicated in figure.

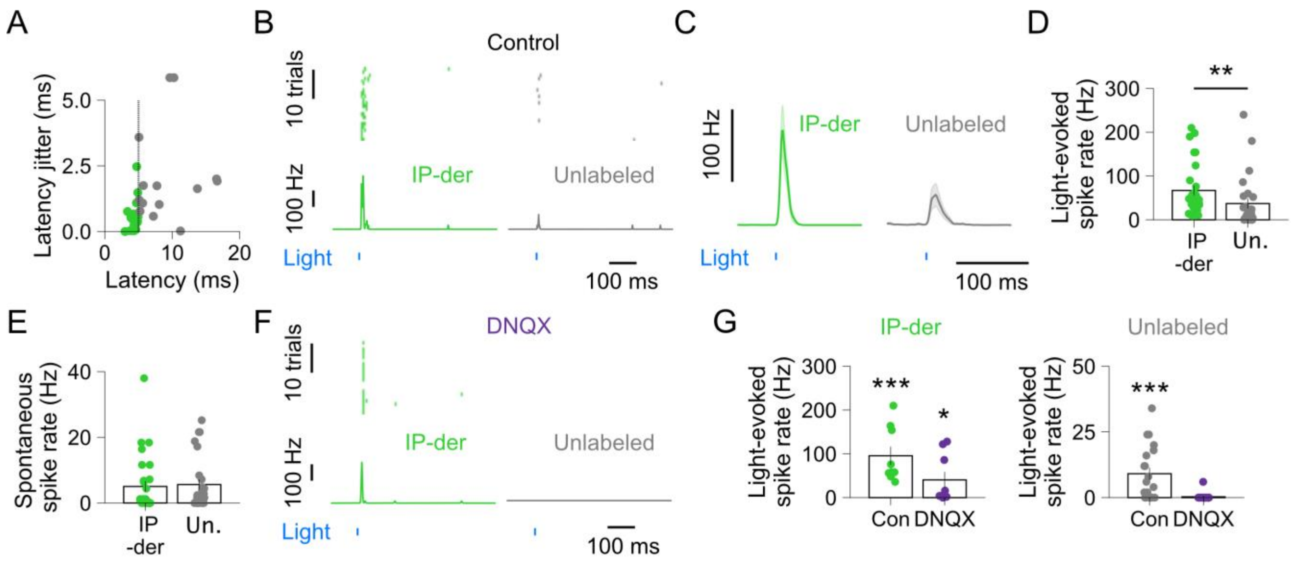

Supplementary Figure 3: *In vivo* identification of IP-derived L4 neurons by optotagging. Related to Figure 2. (**A**) Optotagging was used to distinguish putative IP-derived (i.e., ChR2-YFP expressing) L4 neurons from unlabelled L4 neurons. Based on spike times in response to light pulses (25 repeats, 10 ms pulse), a mean light-evoked spike latency of 5 ms was used as the separation criterion (IP-derived spike latency was 4.34 ± 0.10 ms, unlabelled spike latency was 8.97 ± 1.18 ms, n = 29 and 26). (**B**) Light-evoked spiking of an individual IP-derived neuron (left) and unlabelled neuron (right). (**C**) Mean light-evoked spiking response of a population of IP-derived and unlabelled neurons (n = 29 and 26). (**D**) Light-evoked spiking was higher in IP-derived L4 neurons (IP-derived spike rate was 66.97 ± 11.02 Hz, unlabelled was 37.08 ± 11.56 Hz; n = 29 and 26; p = 0.0018, Mann Whitney U test). (**E**) Spontaneous spike rates did not differ between IP-derived and unlabelled neurons (IP-derived spontaneous spike rate was 5.03 ± 1.60 Hz, unlabelled was 5.62 ± 1.57 Hz; n = 29 and 26; p = 0.484, Mann Whitney U test). (**F**) Example recordings show that after blocking synaptic transmission with the glutamate receptor blocker DNQX, light-evoked spiking was still evident in an optotagged IP-derived neuron but was abolished in a nearby unlabelled neuron. (**G**) Population data revealed that DNQX reduced, but did not abolish, light-evoked responses in IP-derived neurons (control was 95.56 ± 21.01 Hz, DNQX was 40.44 ± 18.35 Hz; p < 0.001 and p = 0.029, one sample Wilcoxon against a median of zero; n = 9). On the other hand, DNQX did abolish light-evoked responses in unlabelled neurons (control was 9.10 ± 2.32 Hz, DNQX was 0.30 ± 0.30 Hz; p < 0.001, p = 0.5, one sample Wilcoxon against a median of zero; n = 20). Data represented as mean ± SEM, n = neurons.

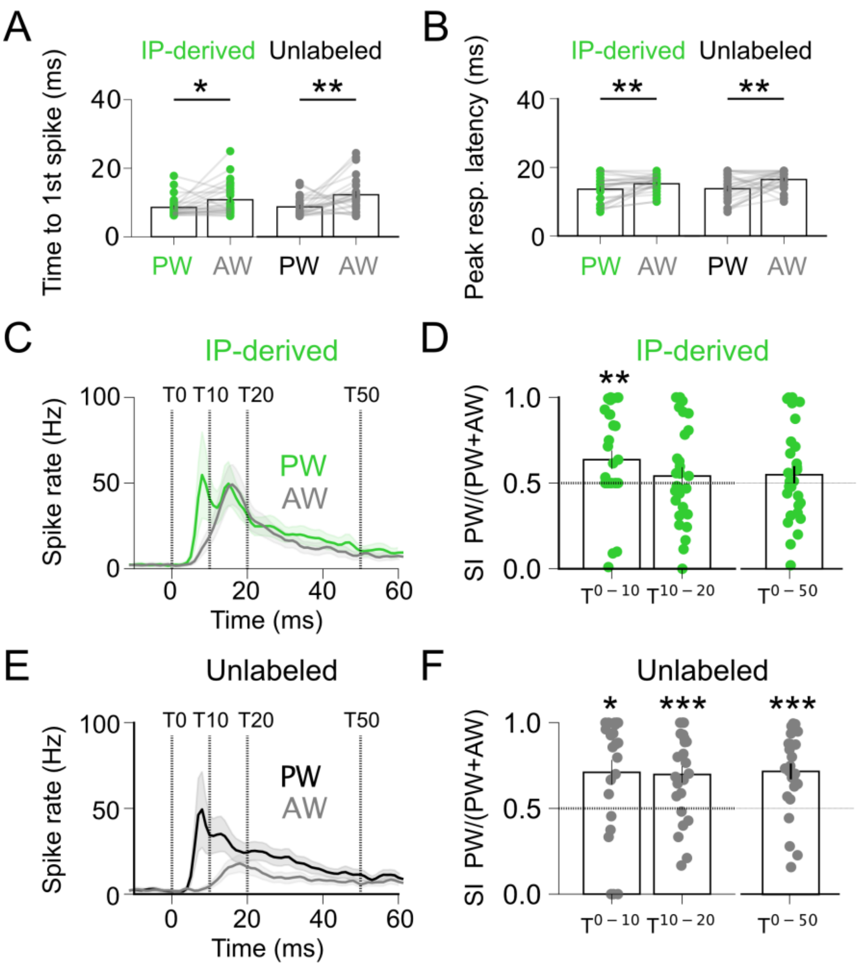

Supplementary Figure 4: The timing of whisker responses suggests that IP-derived L4 neurons receive pronounced recurrent excitatory inputs. Related to Figure 2. **(A)** Consistent with previous work [S10-S12], response latency, estimated as the time to first spike, was shorter following a single deflection of the principal whisker (PW) than following a single deflection of the adjacent whisker (AW), for both IP-derived (n = 24; p = 0.049, Wilcoxon matched pairs) and unlabeled L4 neurons (n = 24; p = 0.0079, Wilcoxon matched pairs). **(B)** Similarly, the peak response latency was shorter following a PW deflection than an AW deflection for both IP-derived (n = 25; p = 0.0017, paired t-test) and unlabeled neurons (n = 26; p = 0.006, Wilcoxon matched pairs). **(C)** Mean spiking responses of IP-derived L4 neurons following PW or AW deflection. Vertical dotted lines (T^{0} to T^{50}) indicate different time points for calculating the selectivity index (SI) following whisker deflection at 0 ms. Shading indicates SEM around the mean. **(D)** In the 0-10 ms immediately following whisker deflection, IP-derived L4 neurons were selective for the PW ($T^{0\text{-}10}$; n = 29; p = 0.0036, Wilcoxon signed-rank against a median of 0.5), consistent with the delayed onset of the AW response. However, 10–20 ms following whisker deflection, IP-derived neurons were no longer selective for the PW ($T^{10\text{-}20}$; n = 29; p = 0.178, one-sample t-test), which was consistent with the SI calculated over the entire 50 ms response window ($T^{0\text{-}50}$; n = 29; p = 0.171, one-sample t-test; **Figure 2F**). Hence, IP-derived responses are only initially selective for the PW (0-10 ms) and then this selectivity is lost, consistent with recurrent activity via thalamus [S10-S12]. **(E)** Mean spiking responses of unlabelled L4 neurons following single PW and AW deflection. **(F)** Unlabelled L4 neurons were selective for the PW during the 0-10 ms immediately following whisker deflection ($T^{0\text{-}10}$; n = 23; p = 0.013, Wilcoxon signed-rank against a median of 0.5). In contrast to IP-derived neurons, however, unlabelled neurons remained selective for the PW at 10–20 ms ($T^{10\text{-}20}$; n = 26; p < 0.001, one-sample t-test), and over the entire 50 ms response window ($T^{0\text{-}50}$; n = 29; p < 0.001 , one-sample t-test; **Figure 2F**), consistent with less recurrent activity via thalamus [S10-S12]. Data represented as mean ± SEM, n = neurons.

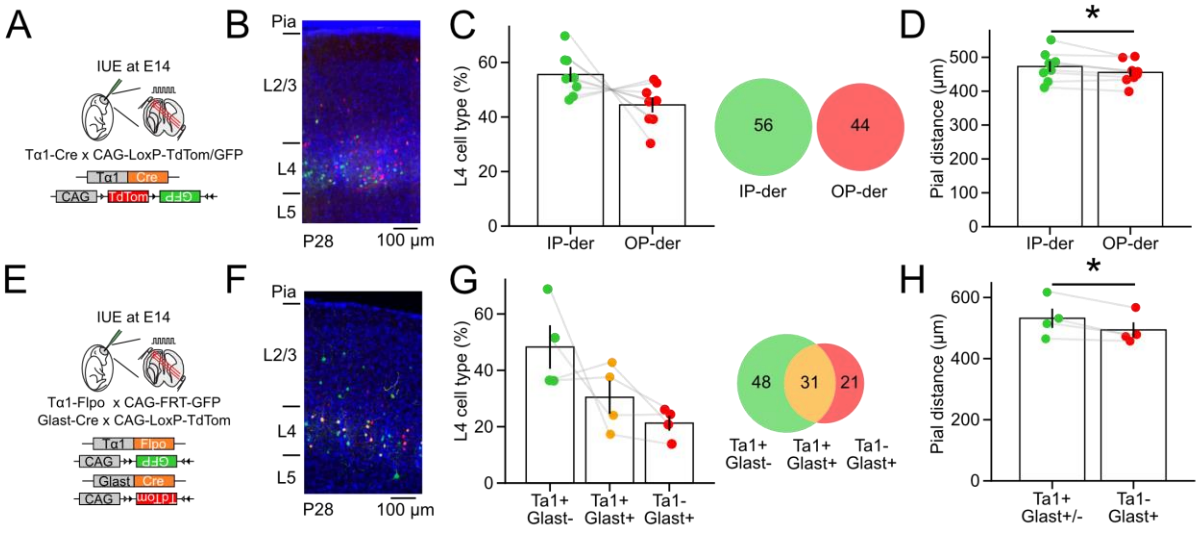

Supplementary Figure 5: The OP-derived L4 neuronal population (i.e. Tα1-negative lineage) includes neurons derived from Glast-positive radial glial cells. Related to Figure 3. (**A**) As in **Figure 3**, animals at E14 underwent IUE of a Tα1-Cre plasmid and a two-colour reporter plasmid that incorporates a flexible excision cassette, where Cre recombination permanently switches expression from tdTomato to GFP. (**B**) At P28 following IUE of the plasmids shown in 'A', 'IP-derived' (i.e. expressing GFP) and 'OP-derived' (i.e. expressing tdTomato) neurons were observed in L4 of S1. (**C**) IP-derived and OP-derived neurons comprised 55.56 ± 2.77 % and 44.44 ± 2.77 % of the labelled neurons in L4, respectively. (**D**) The soma of OP-derived and IP-derived L4 neurons showed overlapping distributions in mature cortex, but the average OP-derived neuron was located closer to the pia (IP-derived was 472.85 ± 15.82 μm, OP-derived was 455.44 ± 11.99 μm, $n = 8$, $p = 0.027$, paired t-test), consistent with the OP population including progenitors that undergo self-renewing divisions over a longer period of embryonic development, as displayed by radial glial cells [S3, S5, S13]. (**E**) To characterise the OP-derived population, animals at E14 underwent IUE of four plasmids encoding Tα1-Flpo, Flpo-dependent GFP, Glast-Cre, and Cre-dependent TdTomato. Previous work has shown that the Glast promoter can be used to label radial glial cells at these ages [S9, S14, S15]. (**F**) At P28 following IUE of the plasmids shown in 'E', L4 neurons derived from either a Tα1-positive/Glast-negative lineage (i.e. expressing GFP only), a Tα1-positive/Glast-positive lineage (i.e. expressing GFP and tdTomato), or a Tα1-negative/Glast-positive lineage (i.e. expressing tdTomato only) were observed in S1. (**G**) The labelled L4 neurons comprised 48.28 ± 7.72 % derived from a Tα1-positive/Glast-negative lineage, 30.44 ± 5.89 % from a Tα1-positive/Glast-positive lineage, and 21.28 ± 2.70 % from a Tα1-negative/Glast-positive lineage. (**H**) On average, neurons derived from the Tα1-negative/Glast-positive lineage were located closer to the pia than neurons derived from the Tα1-positive lineages (Tα1-positive was 532.01 ± 31.73 μm, Tα1-negative/Glast-positive was 493.91 ± 24.90 μm, $n = 4$, $p = 0.043$, paired t-test). These data are consistent with the OP-derived population including neurons derived from Glast-expressing radial glial cells, which undergo self-renewing divisions [S3, S5, S9]. Data represented as mean ± SEM, n = animals. Scale bars are indicated in figure.

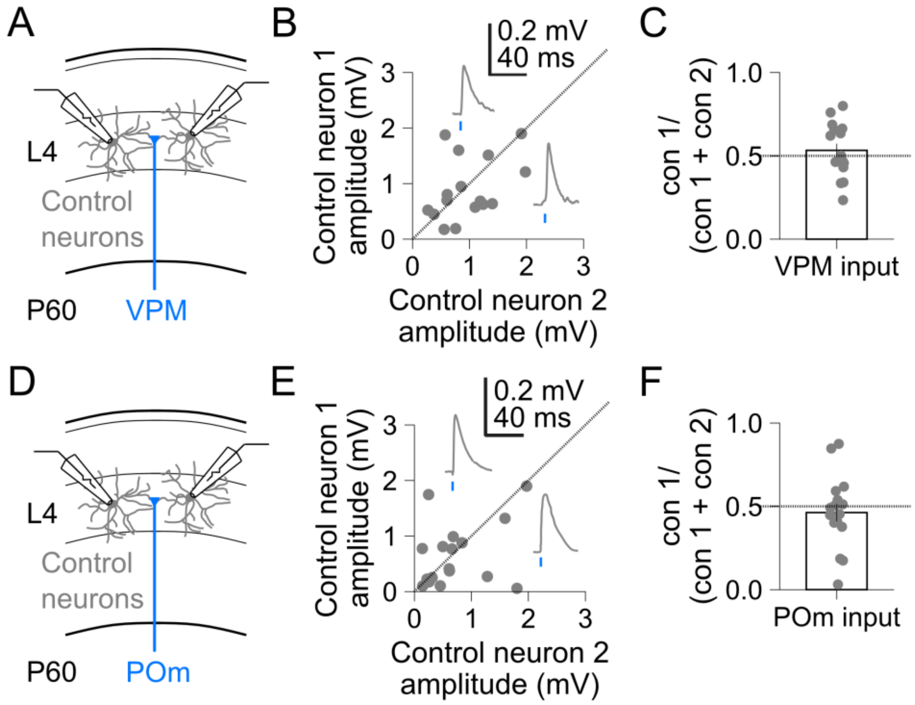

Supplementary Figure 6: No consistent bias of thalamic inputs is observed in pairs of unlabeled L4 neurons. Related to Figure 3. (**A**) Simultaneous whole cell recordings were performed from pairs of unlabeled control L4 neurons at P60, whilst ChR2-GFP-expressing VPM axons were stimulated with light pulses. (**B**) EPSP peak amplitudes for pairs of unlabeled control neurons in response to light stimulation of VPM axons. (**C**) No consistent bias in VPM input was observed under these conditions (0.53 ± 0.04; n = 16; p = 0.214, one sample t-test). (**D**) In a separate set of experiments, simultaneous whole cell recordings were performed from pairs of unlabeled control L4 neurons, whilst ChR2-GFP-expressing POm axons were stimulated. (**E**) EPSP peak amplitudes for pairs of unlabeled control neurons in response to light stimulation of POm axons. (**F**) No consistent bias in POm input was observed under these conditions (0.46 ± 0.05; n = 17; p = 0.241, one sample t-test). Data represented as mean ± SEM, n = neuron pairs.

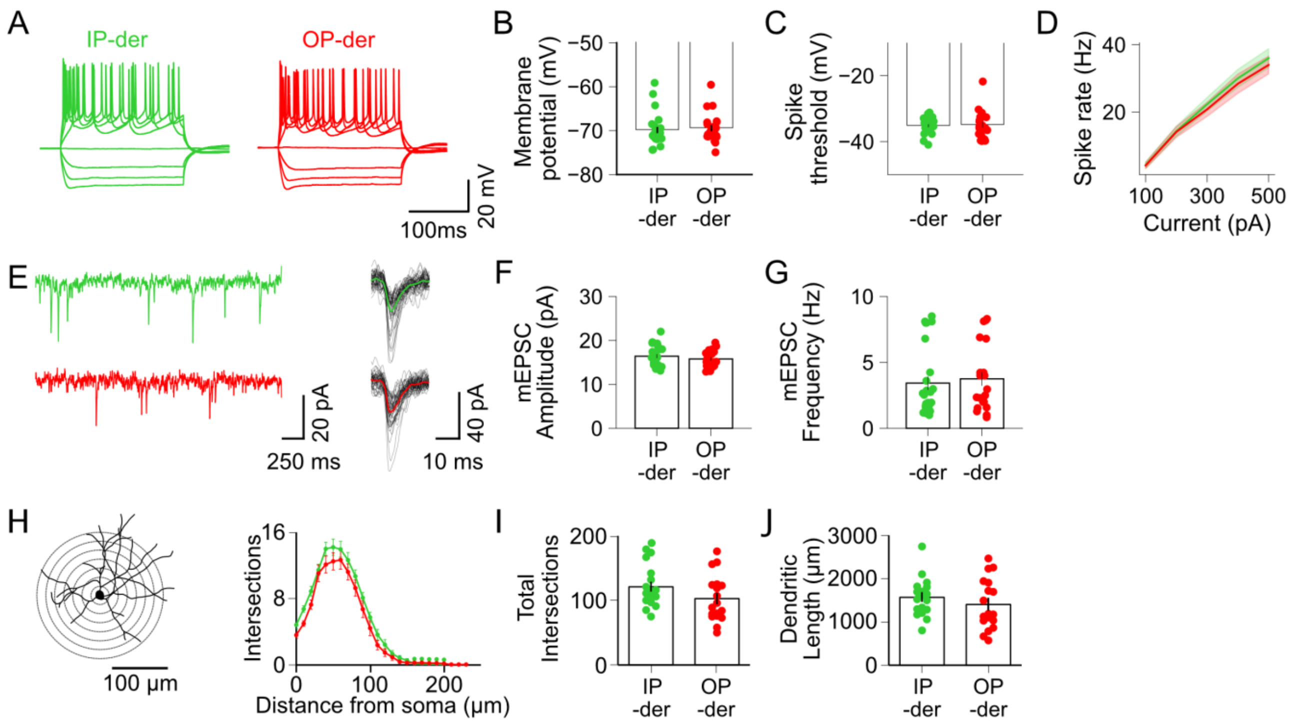

Supplementary Figure 7: Intrinsic electrical, synaptic and morphological properties of IP-derived L4 neurons. Related to Figure 3 and 4. (**A**) Current clamp recordings performed in acute brain slices from an IP-derived and OP-derived L4 neuron, in response to current steps. (**B**) Resting membrane potential was not significantly different (IP-derived was -69.86 ± 0.71 mV, OP-derived was -69.43 ± 0.70 mV; n = 25 and 22; p = 0.482, Mann Whitney U test). (**C**) Spike threshold was not significantly different (IP-derived was -35.18 ± 0.46 mV, OP-derived was -34.89 ± 0.88 mV; n = 25 and 22; p = 0.76, t-test). (**D**) When spike rate in response to five levels of injected current was compared for IP-derived and OP-derived neurons, there was a significant effect of injected current ($F(4,180) = 194.503$, $p<0.001$), no significant effect of cell type ($F(1,45) = 0.18$, $p = 0.673$), and no significant interaction between injected current and cell type ($F(4,180) = 0.248$, $p = 0.769$; n = 25 and 22; Mixed ANOVA). (**E**) Voltage clamp recordings of spontaneous excitatory synaptic currents in an IP-derived and an OP-derived L4 neuron. Composite traces represent the mean of 50 spontaneous events. (**F**) The amplitude of spontaneous excitatory synaptic currents was not different (IP-derived was 16.40 ± 0.45 pA, OP-derived was 15.77 ± 0.40 pA; n = 26 and 22; p = 0.31, t-test). (**G**) The frequency of spontaneous excitatory synaptic currents was not different (IP-derived was 3.55 ± 0.49 Hz, OP-derived was 3.79 ± 0.39 Hz; n=26 and 22; p = 0.45, Mann Whitney U test). (**H**) A Sholl analysis was performed on the dendrites of IP-derived and OP-derived L4 neurons, by counting dendritic intersections at different distances from the soma. (**I**) The total number of intersections was not significantly different (IP-derived was 121 ± 7, OP-derived was 103 ± 8 Hz; n=18 and 18; p = 0.11, t-test). (**J**) The overall dendritic length was not significantly different (IP-derived was 1567 ± 104 µm, OP-derived was 1402 ± 137 µm; n=18 and 18; p = 0.34, t-test). Data represented as mean ± SEM, n = neurons.

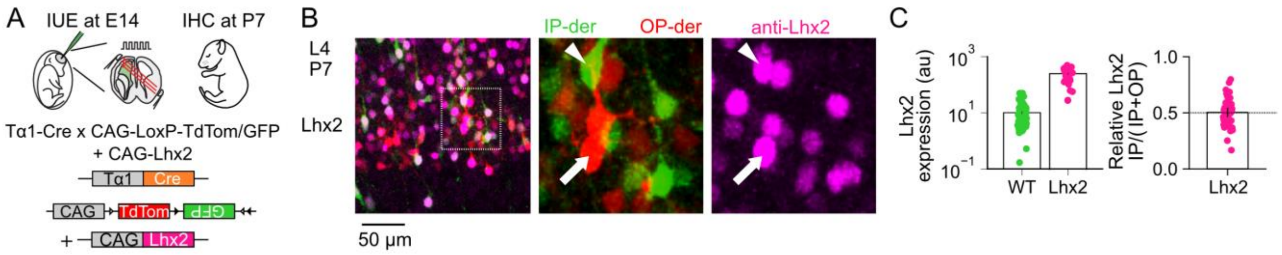

Supplementary Figure 8: An overexpression construct increases Lhx2 levels in L4 neurons labelled by IUE. Related to Figure 5. (**A**) To raise Lhx2 levels, a CAG-Lhx2 overexpression plasmid was delivered with Tα1-Cre and a two-colour Cre-dependent reporter plasmid by IUE. (**B**) Immunohistochemistry at P7 revealed similarly high levels of Lhx2 expression in IP-derived neurons and OP-derived electroporated neurons. (**C**) Quantification confirmed increased levels in IP-derived Lhx2-overexpressing (Lhx2) L4 neurons compared to IP-derived WT L4 neurons (left; WT expression was 10.18 ± 1.16 au, Lhx2 expression was 248.57 ± 19.51 au). Within the Lhx2 electroporated tissue, expression levels were similar in the IP-derived and OP-derived electroporated L4 neurons, such that there was no difference in relative expression (right; 0.50 ± 0.02; n = 40 pairs, p = 0.423, one sample t-test). Data represented as mean ± SEM, n = neurons. Scale bars are indicated in figure.

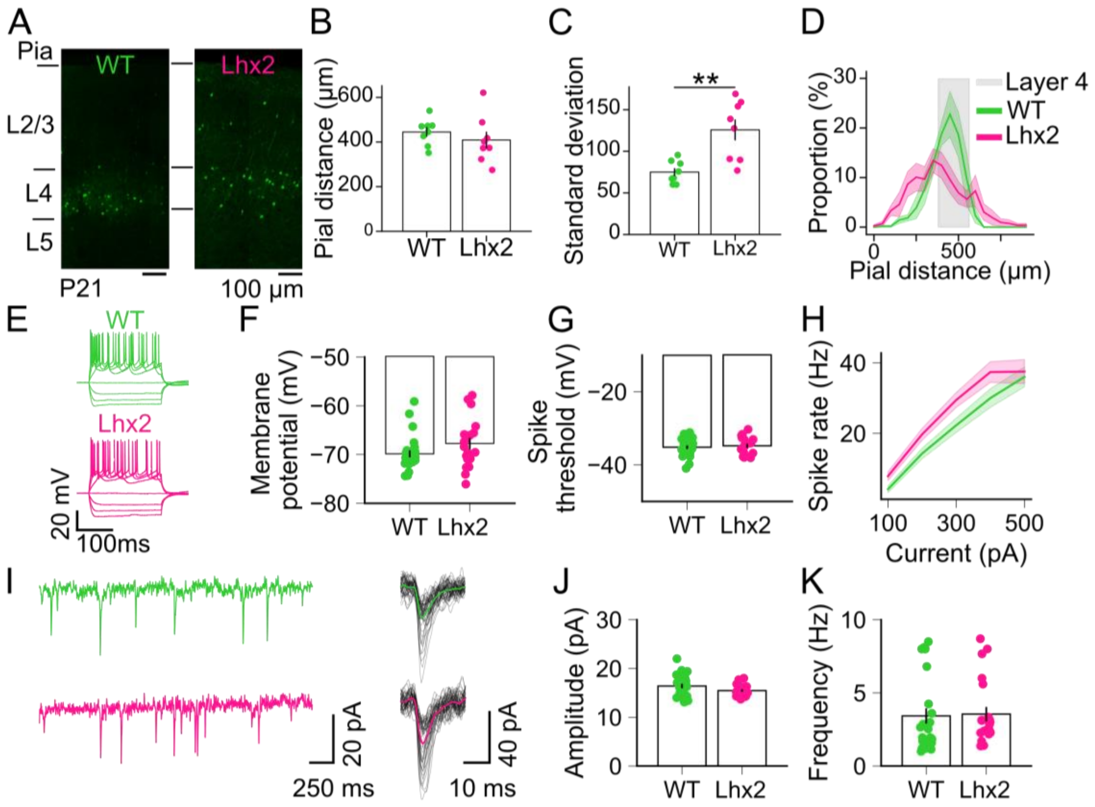

Supplementary Figure 9: Effects of increased Lhx2 on properties of IP-derived L4 neurons. Related to Figure 5. (**A**) IP-derived wild-type (WT) neurons (left) at P21, labeled via IUE of the Tα1-Cre plasmid and a reporter plasmid at E14. IP-derived Lhx2-overexpressing neurons (Lhx2; right) at P21, labeled via IUE of the Tα1-Cre plasmid, a Cre-dependent reporter plasmid, and a CAG-Lhx2 plasmid to increase Lhx2 expression levels in the electroporated neurons. (**B**) The soma of electroporated WT and Lhx2 IP-derived neurons were located at similar mean distances from the pia mater (WT depth 445.07 ± 20.92 µm, Lhx2 depth 409.29 ± 37.63 µm, n = 8 animals per condition, p = 0.42, t-test). (**C**) The standard deviation of distances from pia was higher for Lhx2 IP-derived neurons than WT IP-derived neurons (WT was 75.02 ± 4.77, Lhx2 was 125.76 ± 12.58, n = 8 animals per condition, p = 0.002, t-test), indicating greater variability in the distances migrated by Lhx2 IP-derived neurons. (**D**) Distribution of pial distances of IP-derived neurons for WT and Lhx2 conditions. (**E**) Current clamp recordings from an IP-derived WT and Lhx2 L4 neuron in response to current steps. (**F**) Recordings did not reveal a statistical difference in resting membrane potential between IP-derived WT and Lhx2 neurons (WT, -69.86 ± 0.71 mV; Lhx2, -67.74 ± 1.16 mV; n = 25 and 19; p = 0.084, Mann Whitney U test). (**G**) Recordings did not reveal a statistical difference in spike threshold (WT, -35.18 ± 0.46 mV; Lhx2, -34.60 ± 0.84 mV; n = 25 and 14; p = 0.51, t-test). (**H**) When spike rate in response to five levels of injected current was compared for WT and Lhx2 IP-derived neurons, there was a significant effect of injected current (F(4,168) = 168.523, p<0.001), no significant effect of cell type (F(1,42) = 3.514, p = 0.067), and no significant interaction between injected current and cell type (F(4,168) = 1.634, p = 0.206; n = 25 and 19; Mixed ANOVA). (**I**) Voltage clamp recordings of spontaneous excitatory synaptic currents in an IP-derived WT and Lhx2 L4 neuron. (**J**) Recordings did not reveal a statistical difference in the amplitude of spontaneous excitatory synaptic currents between IP-derived WT and Lhx2 neurons (WT, 16.40 ± 0.45 pA; Lhx2, 15.47 ± 0.25 pA; n = 26 and 21; p = 0.09, t-test). (**K**) Recordings did not reveal a statistical difference in the frequency of spontaneous excitatory synaptic currents (WT, 3.43 ± 0.52 Hz; Lhx2, 3.55 ± 0.49 Hz; n = 26 and 21; p = 0.36, Mann Whitney U test). Data represented as mean ± SEM, n = animals or neurons. Scale bars are indicated in figure.

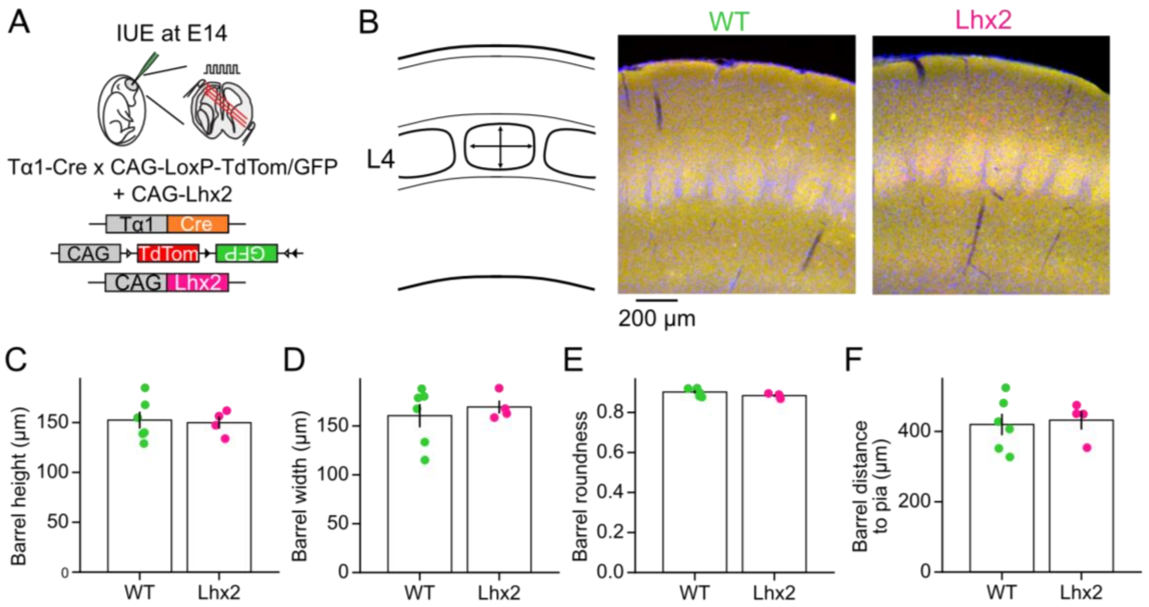

Supplementary Figure 10: *In utero* electroporation of Lhx2 does not affect the overall structure of barrels. Related to Figure 5. (**A**) Animals at E14 underwent IUE of a Tα1-Cre, a two-colour Cre-dependent reporter, and a CAG-Lhx2 overexpression plasmid. (**B**) At P21, barrels from electroporated animals were visualised and compared to those from animals that did not receive the Lhx2 overexpression plasmid (WT). (**C**) Barrel height was not different between Lhx2 and WT animals (Lhx2 was 149.88 ± 6.13 µm, WT was 152.49 ± 8.53 µm, n = 4 and 6, p = 0.829, t-test). (**D**) Barrel width was not different between Lhx2 and WT animals (Lhx2 was 169.41 ± 6.67 µm, WT was 160.53 ± 12.00 µm, n = 4 and 6, p = 0.592, t-test). (**E**) Barrel roundness was not different between Lhx2 and WT animals (Lhx2 was 0.88 ± 0.01, WT was 0.90 ± 0.01, n = 6 and 4, p = 0.142, t-test). (**F**) Distance from barrel centre to pia was not different between Lhx2 and WT animals (Lhx2 was 432.04 ± 26.74 µm, WT was 419.77 ± 30.55 µm, n = 4 and 6, p = 0.786, t-test). Data represented as mean ± SEM, n = animals. Scale bars are indicated in figure.

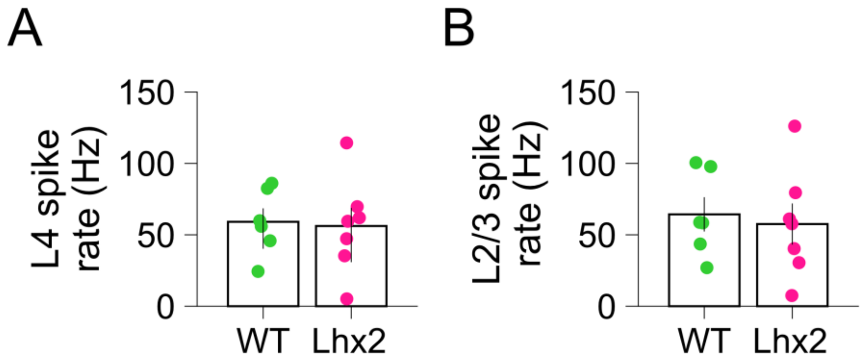

Supplementary Figure 11: Overall spiking activity during the rhythmic whisker stimulation protocol was comparable across conditions. Related to Figure 7. (**A**) Multiunit spiking activity in L4 during the rhythmic whisker stimulation (RWS; 8 Hz for 60 s) was similar for animals with IP-derived WT or Lhx2 L4 neurons (WT was 59.10 ± 9.43 Hz, Lhx2 was 56.17 ± 12.66 Hz; n = 6 and 7; p = 0.86, t-test). (**B**) Multiunit spiking activity in L2/3 during RWS was not different for animals with IP-derived WT or Lhx2 L4 neurons (WT was 64.31 ± 12.00 Hz, Lhx2 was 57.53 ± 14.42; n = 6 and 7; p = 0.73, t-test). Data represented as mean ± SEM, n = animals.

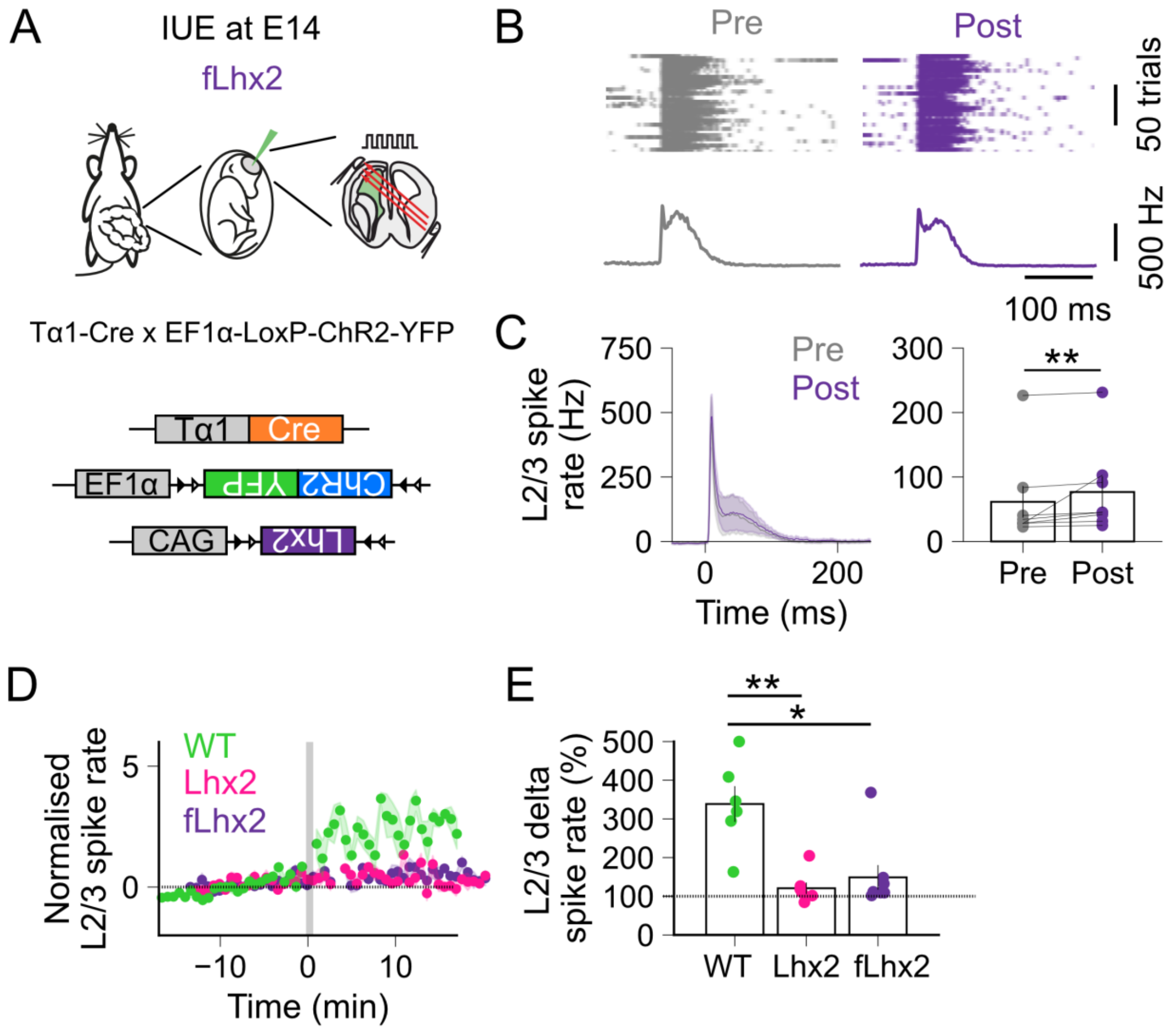

Supplementary Figure 12: Selectively increasing Lhx2 levels in IP-derived neurons reduces sensory-evoked plasticity in L2/3. Related to Figure 7. **(A)** A floxed CAG-Lhx2 plasmid was generated that only expresses Lhx2 upon Cre recombination. Animals at E14 underwent IUE of a Tα1-Cre, a floxed ChR2-YFP, and the floxed CAG-Lhx2 plasmid, in order to restrict Lhx2 overexpression to IP-derived neurons (referred to as 'fLhx2' animals). **(B)** Once the fLhx2 animals reached P28, they were subjected to the rhythmic whisker stimulation (RWS) protocol. Raster plots (top) and PSTHs (bottom) show multiunit activity in L2/3. Responses to single whisker deflections (0.1 Hz) are shown before (pre) and after (post) RWS. **(C)** Mean (left) and separate (right) population data reveals that RWS induced a mild, statistically significant potentiation of L2/3 activity in fLhx2 animals ($n = 8$; $p = 0.008$, Wilcoxon matched pairs). Shading indicates SEM around mean. (**D**) Normalised L2/3 multiunit activity relative to the time of RWS. Each data point is mean of five whisker deflections delivered at 0.1 Hz. (**E**) The RWS-induced increase in spike rate was significantly higher in WT animals than in either Lhx2 or fLhx2 animals (n = 6, 7 and 8 animals; $p = 0.006$, Kruskall Wallis test; WT vs. Lhx2, $p < 0.01$; WT vs. fLhx2, $p < 0.05$; Lhx2 vs. fLhx2, $p > 0.05$; Dunn's test). Thus, restricting increased Lhx2 expression to IP-derived neurons is sufficient to reduce sensory-evoked plasticity in L2/3. Data represented as mean ± SEM, n = animals.

Supplemental Information References

S1. Okun, M., Steinmetz, N.A., Cossell, L., Iacaruso, M.F., Ko, H., Barthó, P., Moore, T., Hofer, S.B., Mrsic-Flogel, T.D., Carandini, M., et al. (2015). Diverse coupling of neurons to populations in sensory cortex. Nature 521, 511–515. 10.1038/nature14273.

S2. Noctor, S.C., Martínez-Cerdeño, V., and Kriegstein, A.R. (2008). Distinct behaviors of neural stem and progenitor cells underlie cortical neurogenesis. J Comp Neurol 508, 28–44. 10.1002/cne.21669.

S3. Englund, C. et al. Pax6, Tbr2, and Tbr1 are expressed sequentially by radial glia, intermediate progenitor cells, and postmitotic neurons in developing neocortex. J Neurosci 25, 247–251 (2005).

S4. Kawaguchi, A., Ikawa, T., Kasukawa, T., Ueda, H.R., Kurimoto, K., Saitou, M., and Matsuzaki, F. (2008). Single-cell gene profiling defines differential progenitor subclasses in mammalian neurogenesis. Development 135, 3113–3124. 10.1242/dev.022616.

S5. Noctor, S.C., Martínez-Cerdeño, V., Ivic, L., and Kriegstein, A.R. (2004). Cortical neurons arise in symmetric and asymmetric division zones and migrate through specific phases. Nat Neurosci 7, 136–144. 10.1038/nn1172.

S6. Wu, S.-X. et al. Pyramidal neurons of upper cortical layers generated by NEX-positive progenitor cells in the subventricular zone. Proc Natl Acad Sci U S A 102, 17172–17177 (2005).

S7. Tyler, W. A. & Haydar, T. F. Multiplex genetic fate mapping reveals a novel route of neocortical neurogenesis, which is altered in the Ts65Dn mouse model of Down syndrome. J Neurosci 33, 5106–5119 (2013).

S8. Gal, J. S. Molecular and Morphological Heterogeneity of Neural Precursors in the Mouse Neocortical Proliferative Zones. Journal of Neuroscience 26, 1045–1056 (2006).

S9. Stancik, E. K., Navarro-Quiroga, I., Sellke, R. & Haydar, T. F. Heterogeneity in Ventricular Zone Neural Precursors Contributes to Neuronal Fate Diversity in the Postnatal Neocortex. Journal of Neuroscience 30, 7028–7036 (2010).

S10. Diamond, M.E., Armstrong-James, M., Budway, M.J., and Ebner, F.F. (1992). Somatic sensory responses in the rostral sector of the posterior group (POm) and in the ventral posterior medial nucleus (VPM) of the rat thalamus: Dependence on the barrel field cortex. J. Comp. Neurol. 319, 66–84. 10.1002/cne.903190108.

S11. Armstrong-James, M., Fox, K. & Das-Gupta, A. Flow of excitation within rat barrel cortex on striking a single vibrissa. J Neurophysiol 68, 1345–1358 (1992).

S12. Armstrong-James, M. & Fox, K. Spatiotemporal convergence and divergence in the rat S1 ‘barrel’ cortex. J Comp Neurol 263, 265–281 (1987).

S13. Gao, P. et al. Deterministic progenitor behavior and unitary production of neurons in the neocortex. Cell 159, 775–788 (2014).

S14. Shibata, T. et al. Glutamate transporter GLAST is expressed in the radial glia-astrocyte lineage of developing mouse spinal cord. J Neurosci 17, 9212–9219 (1997).

S15. Campbell, K. & Götz, M. Radial glia: multi-purpose cells for vertebrate brain development. Trends Neurosci 25, 235–238 (2002).
